# A replicating RNA vaccine confers protection in a rhesus macaque model of Crimean-Congo hemorrhagic fever

**DOI:** 10.1038/s41541-024-00887-z

**Published:** 2024-05-20

**Authors:** David W. Hawman, Shanna S. Leventhal, Kimberly Meade-White, Amit Khandhar, Justin Murray, Jamie Lovaglio, Carl Shaia, Greg Saturday, Troy Hinkley, Jesse Erasmus, Heinz Feldmann

**Affiliations:** 1 Laboratory of Virology, Division of Intramural Research, National Institute of Allergy and Infectious Diseases, National Institutes of Health, Rocky Mountain Laboratories, Hamilton, MT 59840 USA; 2 HDT Bio, Seattle, WA 98102 USA; 3 Rocky Mountain Veterinary Branch, Division of Intramural Research, National Institute of Allergy and Infectious Diseases, National Institutes of Health, Rocky Mountain Laboratories, Hamilton, MT 59840 USA

**Keywords:** RNA vaccines, Virology

## Abstract

Crimean-Congo hemorrhagic fever (CCHF) is a tick-borne febrile illness with a wide geographic distribution. In recent years the geographic range of Crimean-Congo hemorrhagic fever virus (CCHFV) and its tick vector have increased, placing an increasing number of people at risk of CCHFV infection. Currently, there are no widely available vaccines, and although the World Health Organization recommends ribavirin for treatment, its efficacy is unclear. Here we evaluate a promising replicating RNA vaccine in a rhesus macaque (*Macaca mulatta)* model of CCHF. This model provides an alternative to the established cynomolgus macaque model and recapitulates mild-to-moderate human disease. Rhesus macaques infected with CCHFV consistently exhibit viremia, detectable viral RNA in a multitude of tissues, and moderate pathology in the liver and spleen. We used this model to evaluate the immunogenicity and protective efficacy of a replicating RNA vaccine. Rhesus macaques vaccinated with RNAs expressing the CCHFV nucleoprotein and glycoprotein precursor developed robust non-neutralizing humoral immunity against the CCHFV nucleoprotein and had significant protection against the CCHFV challenge. Together, our data report a model of CCHF using rhesus macaques and demonstrate that our replicating RNA vaccine is immunogenic and protective in non-human primates after a prime-boost immunization.

## Introduction

Crimean-Congo hemorrhagic fever (CCHF), caused by the CCHF virus (CCHFV) is a tick-borne virus that can cause a severe hemorrhagic disease in infected humans. Similar to other viral hemorrhagic fevers, CCHF begins as non-specific fever, myalgia, nausea, diarrhea, and general malaise^1,2^. In some, this disease can rapidly progress to hemorrhagic manifestations, and case fatality rates can be as high as 30–40% in some regions^1,2^. Currently, the only widely used therapy is ribavirin but efficacy in both humans and animal models is conflicting and suggestive of poor efficacy when treatment is started later in infection^2,3^. Besides an inactivated preparation of CCHFV grown in mouse brains used as a vaccine in Bulgaria^4^, there are no approved vaccines for CCHF, and prevention is limited to control of exposure to infected ticks and livestock.

We have previously evaluated a replicating RNA (repRNA) vaccine for CCHFV in a lethal mouse challenge model^5^. This vaccine is based on an alphavirus replicon system^6^ in which the structural proteins of the Venezuelan equine encephalitis virus, strain TC-83, are replaced with a gene-of-interest. This results in an RNA capable of self-amplification, leading to dose sparing and mimicking an authentic viral infection while being unable to spread from the initially transfected cell, conferring a significant safety margin. Delivery of the repRNA is accomplished by complexing the RNA with a cationic nanocarrier called LION^7^ that (1) may induce less systemic inflammation than current lipid nanoparticles used for many mRNA vaccines^8^, (2) has been manufactured under current good manufacturing practices, (3) has demonstrated safety and immunogenicity in humans^9,10^, and (4) is the basis for a product that achieved emergency use authorization in India^11^. In our mouse studies, we evaluated repRNA expressing either the CCHFV nucleoprotein (NP, repNP) or the full-length CCHFV glycoprotein precursor (GPC, repGPC). Surprisingly, we found that our repNP vaccine could confer robust protection on its own after a single low-dose immunization, and protection correlated with a non-neutralizing antibody response^5^. In contrast, our repGPC vaccine was only partially protective and was associated with strong CCHFV-specific T-cell responses^5^. However, the inclusion of both antigens leads to optimal control of the challenge^5^. In addition to CCHFV, this repRNA platform has been evaluated in pre-clinical models for SARS-CoV-2 (COVID-19), Zika virus, and tuberculosis^7,12–14^.

We and others have previously established a non-human primate (NHP) model of CCHF using cynomolgus macaques (*Macaca fascicularis*) (CM)^15–20^. Disease in this model is mild-to-moderate with severe disease, as reported in the first description of the model^15^, rarely observed in subsequent studies. Here we established an alternative model of CCHF using rhesus macaques (*Macaca mulatta*) (RM) infected with a strain of CCHFV serially passaged in CM. We show that this passaged strain of CCHFV causes viremia and mild disease consistently in RM providing an additional pre-clinical model for evaluation of CCHFV vaccines and therapeutics. Prime-boost vaccination of RM with repNP and repGPC conferred significant protection against the CCHFV challenge in both models. Protection correlated with humoral immunity primarily directed against the CCHFV NP. Cumulatively, our data establish an RM model of CCHF and demonstrate the protective efficacy of our repRNA vaccine.

## Results

### NHP-passaged CCHFV causes mild-to-moderate disease in RM

Despite the initial report of the CM model of CCHF in which animals developed severe and even terminal disease^15^, the disease in subsequent studies has been mild-to-moderate with only occasional terminal disease. In an attempt to generate a strain of CCHFV that consistently caused severe disease in CM, we passaged CCHFV from the liver of a placebo-treated CM that reached terminal disease on CCHFV on day 5 post-infection (PI)^17^ four times in the livers of naïve, untreated CM. After the last passage, the virus was amplified once in SW13 cells to generate CM-passaged CCHFV (CMP-CCHFV) and sequenced. Sequencing identified one coding mutation in the consensus S segment, two coding mutations in the NSm and Gc of the M segment, and 4 coding mutations in the L segment (Table 1).

**Table 1:** Mutations found in CMP-CCHFV

Segment	Nucleotide change	Amino acid change
S	A563G	NP Gly184>Ser, NSs Pro93>Leu
M	G2802A	Cys917>Tyr (NSm)
	A3209G	Met1053>Val (Gc)
L	T1143A	Synonymous
	A2420G	Asp796Gly
	T8091C	Synonymous
	S8186C	Ser2718Thr
	A10784G	Gln3584Arg
	G11194A	Ala3721Thr
	A11574G	Synonymous

**Fig. 1:**
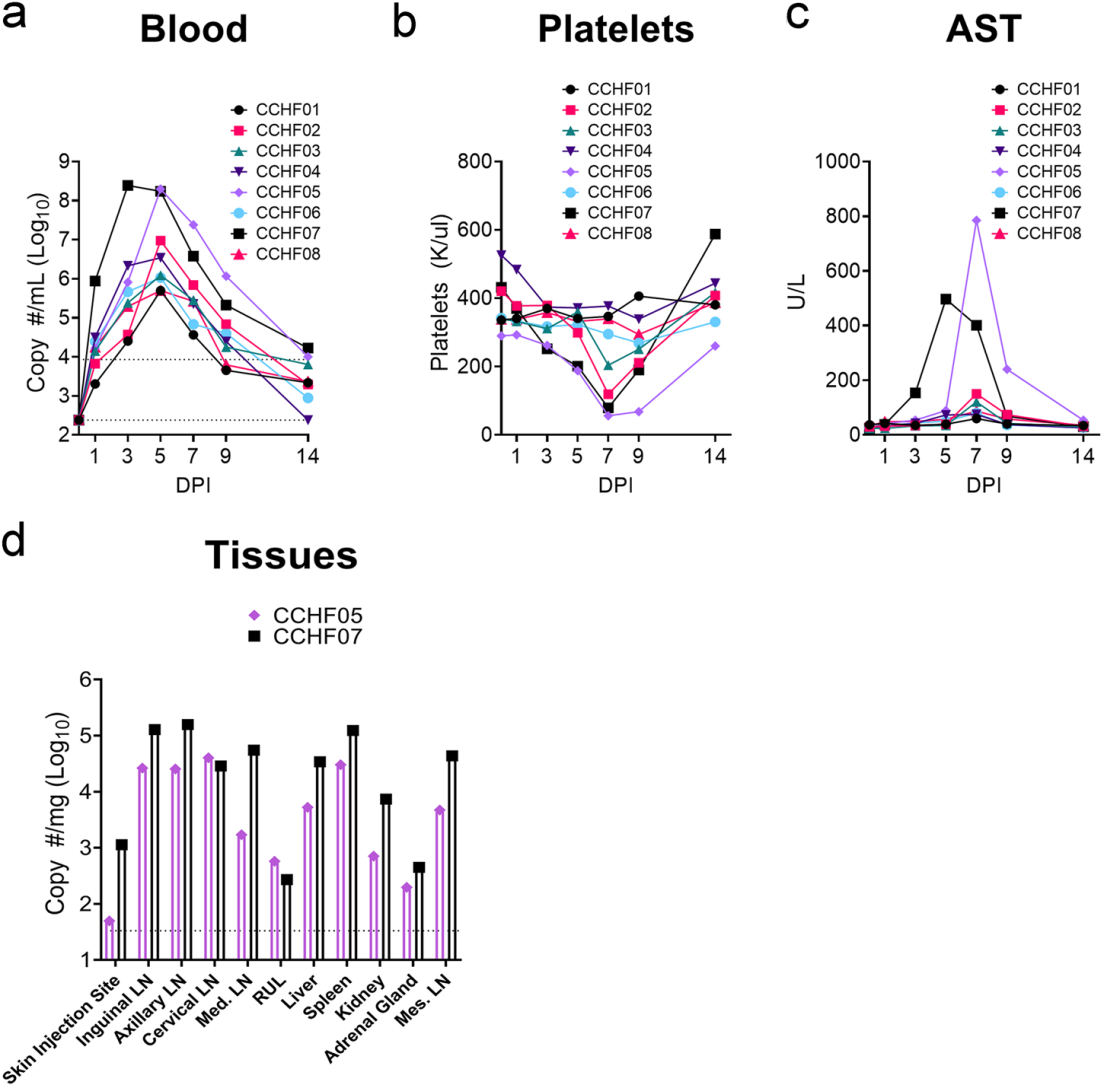
CMP-CCHFV causes mild-to-moderate disease in RM. Rhesus macaques were infected with 10^5^ TCID50 of CMP-CCHFV via the combined IV and SQ routes. Viral RNA (**a**) and platelet count in the blood (**b**) were quantified along with serum AST levels (**c**). Two animals that showed the highest viremia, greatest decrease in platelets, and highest AST levels were euthanized on day 14 for evaluation of viral RNA loads in several tissues (**d**). The remaining animals were released from the study, and no further samples were collected. **a** Upper dashed line indicates the limit of quantitation and the lower dashed line indicates the limit of detection. **d** Dashed line indicates the limit of quantitation.

During the SARS-CoV-2 pandemic, NHPs, especially CM, became difficult to obtain, and alternative NHP models of CCHF would enable continued pre-clinical development of countermeasures for CCHFV. We retained access to RM, and although previous infections of RM with human isolates of CCHFV resulted in no overt disease^15^, we hypothesized that CMP-CCHFV may have acquired mutations that could cause reliable disease in macaques in general. To evaluate whether CMP-CCHFV infection of RM would provide an alternative model of CCHF, we infected a cohort of Indian origin, male and female RM with 100,000 TCID50 of CMP-CCHFV via combined subcutaneous (SQ) and intravenous (IV) routes as previously established for CM^15^. RM infected with CMP-CCHFV developed viremia that peaked on day 3 PI for one animal and day 5 PI for the remainder (Fig. 1a). Viremia correlated with a mild decrease in platelets in three animals (Fig. 1b) and increased liver enzyme aspartate aminotransferase (AST) in two animals (Fig. 1c). The two animals exhibiting the highest viremia and liver enzymes were euthanized on day 14 PI and viral loads in a variety of tissues measured by RT-qPCR. Viral RNA was detected in all tissues evaluated (Fig. 1d). Due to the remaining animals exhibiting no signs of clinical disease, tissues were not collected. The complete hematological and blood chemistry parameters are provided in Supplemental Table 1. Cumulatively, these data demonstrated that CMP-CCHFV infection of RM resulted in consistent viremia and mostly mild disease with occasional moderate disease.

### repNP + repGPC is immunogenic in RM

**Fig. 2:**
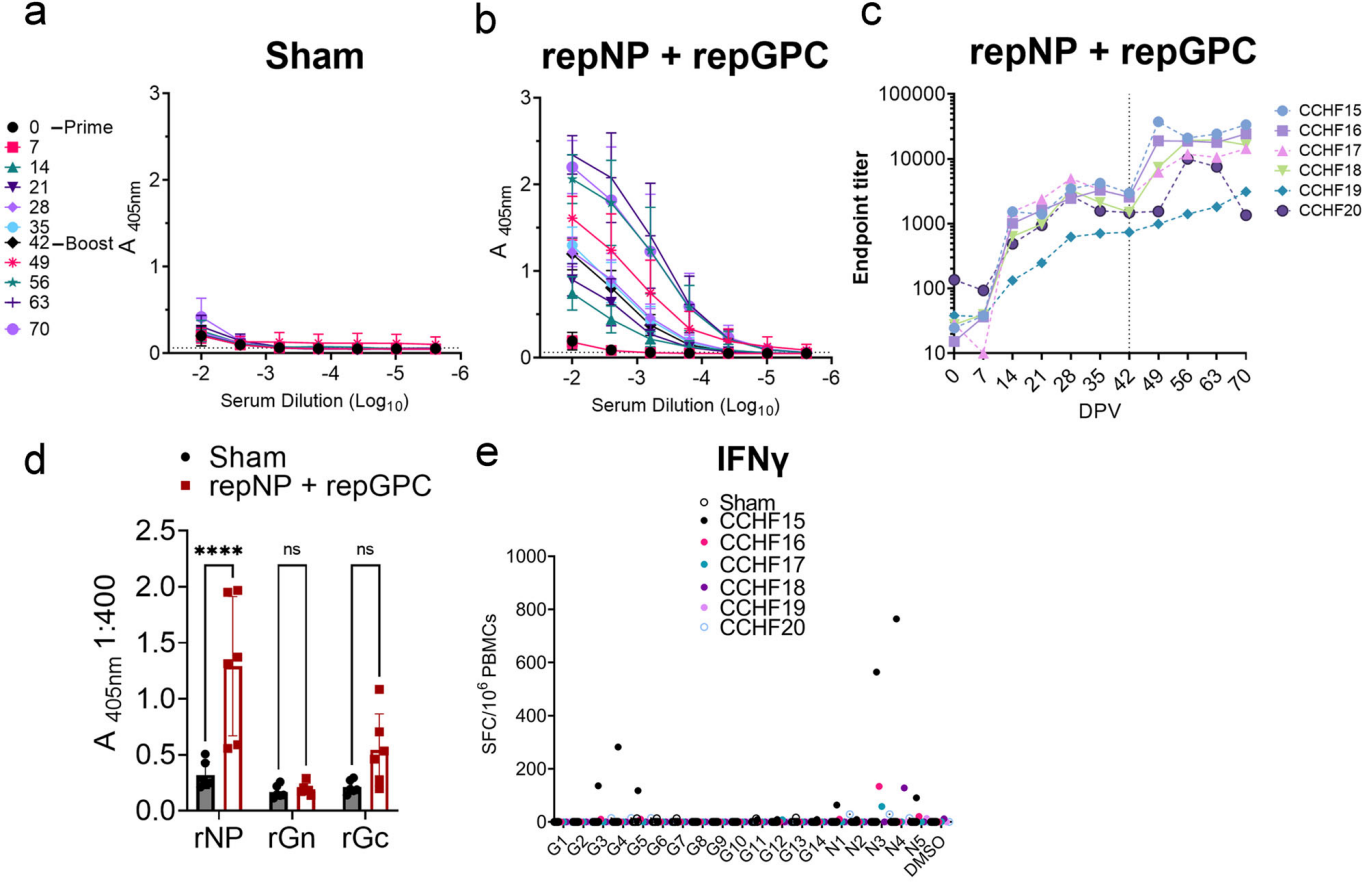
repNP + repGPC is immunogenic in RM. Groups of RM were sham-vaccinated or vaccinated with repNP + repGPC. CCHFV-specific IgG to whole virus antigen was measured at indicated day post-prime vaccination (DPV) (**a**–**c**). **c** is data from (**b**) but individual endpoint titers for each repNP + repGPC animals is shown. **d** IgG responses to specific recombinant antigens were measured by ELISA. **e** An IFNγ ELISpot was performed to measure CCHFV-specific responses to peptides spanning the GPC (G1–14) or NP (N1–5). **d** *P* values calculated with a two-way ANOVA with Sidak’s multiple comparisons test. ns *P* > 0.05, ****P < 0.0001. **a**, **b**, **d** Data shown as mean plus standard deviation.

We then evaluated whether our replicating RNA vaccine^5^ could confer protection against viral replication in this model following a prime-boost vaccination schedule. Indian origin, male and female, RM (*n* = 6) were vaccinated with 25 μg each of our repRNA expressing either the CCHFV NP (repNP) or the GPC (repGPC). Vaccine antigens were based on the Hoti strain of CCHFV^21^. As a control group, RM (*n* = 6) were vaccinated with 25 μg of a repRNA expressing an irrelevant antigen (Enterovirus D68 capsid). The sex, age, and grouping of the RM are listed in Supplemental Table 2. As measured by whole-Hoti virion ELISA, sham-vaccinated animals did not develop a CCHFV-specific antibody response (Fig. 2a), while RM vaccinated with repNP + repGPC rapidly developed CCHFV-specific IgG within 2 weeks post-prime vaccination (PV) (Fig. 2b and c). Titers continued to increase until 4 weeks (day −28 (Fig. 2b and c). Animals were boosted with identical immunizations 6 weeks PV, and except for animal CCHF20, CCHFV-specific IgG titers increased until the time of challenge (Fig. 2b and c). Strikingly, CCHF20 exhibited a substantial drop in CCHFV-specific titers from week 3 to week 4 post-boost, back to titers seen prior to boosting (Fig. 2c). We further investigated the CCHFV-specific antibody response using recombinant NP, Gn and Gc antigens from CCHFV strain 10200 on serum collected at the time of challenge (Day 70 PV) (Fig. 2d). Compared to sham-vaccinated animals, we measured significant antibody responses against the CCHFV NP but not Gc or Gn (Fig. 2d) although there was a trend towards increased antibody response against Gc.

We also evaluated CCHFV-specific T-cell responses on PBMCs collected 2 weeks after animals were boosted using overlapping peptides spanning the entire NP and GPC. In contrast to our studies in mice^5^, we measured little CCHFV-specific T-cell responses in most repNP + repGPC vaccinated RM (Fig. 2e). One animal, CCHF15, had robust T-cell responses (64–764 SFCs/10^6^ PBMCs) against pools 3–5 in the GPC and pools 1,3,4 and 5 in the NP (Fig. 2e). Three additional animals developed modest responses against pools 3 or 4 of the NP (Fig. 2e). Although we cannot exclude the presence of tissue-resident T-cells, these data suggest that repNP + repGPC vaccination elicits primarily humoral immunity in RM and that humoral immunity is largely directed against the CCHFV NP.

### repNP + repGPC protects RM against CCHFV challenge

**Fig. 3:**
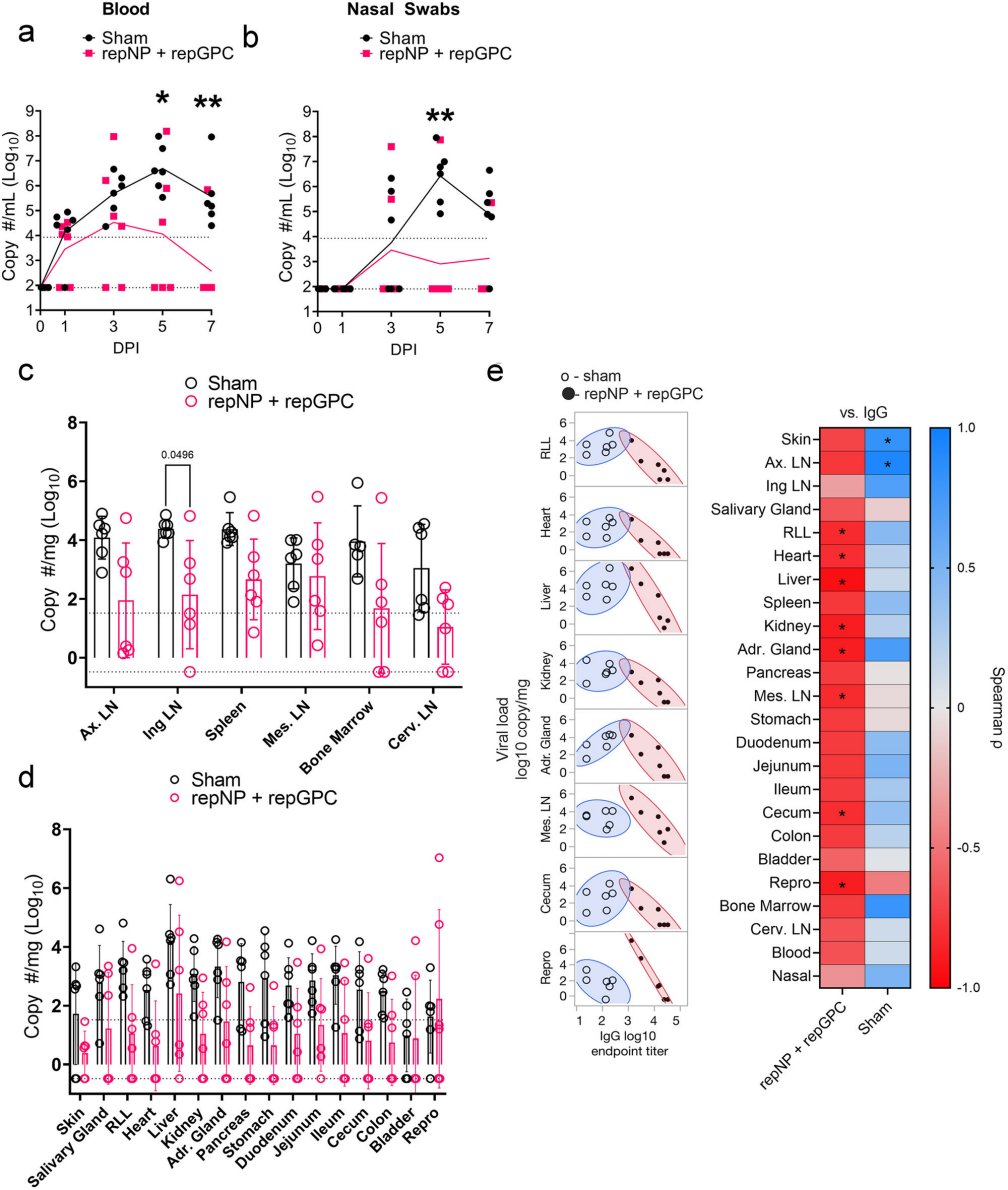
Protection in RM correlates with CCHFV-specific IgG. Viral RNA in the blood (**a**), nasal swabs (**b**), lymphoid tissues (**c**), and non-lymphoid tissues (**d**) was quantified using qRT-PCR. Upper dashed line indicates the limit of quantitation, and the lower dashed line indicates the limit of detection. **e** Spearman correlation between antibody endpoint titers at time of challenge (Day 0 PI) and viral loads at time of necropsy (Day 7 PI) or area under the curve for blood and nasal swab. Tissues with significant correlation are shown in the left panel, and coefficients for all tissues are shown in the right panel. **a**–**d** *P* values calculated using a two-way ANOVA with Sidak’s multiple comparisons test, and comparisons with *P* > 0.05 are not shown. **e** *P* values calculated using Spearman correlation. *P < 0.05, **P < 0.01. **c**, **d** Bar shows mean with error bars representing standard deviation.

Vaccinated RM were challenged with 100,000 TCID50 of CMP-CCHFV as before. Exams were conducted on days 0, 1, 3, 5, and 7 PI and animals were euthanized on day 7 PI for evaluation of viral loads in a variety of tissues. In the blood, compared to sham-vaccinated animals, we measured significantly less viral RNA at day 5 and 7 PI (Fig. 3a) suggesting vaccination conferred more rapid control of viremia. Similarly, in the nasal swabs we also measured significantly less RNA in the repNP + repGPC vaccinated group compared to sham-vaccinated on day 5 PI (Fig. 3b). In tissues, three animals in the repNP + repGPC group had viral RNA below our LoD in most tissues (CCHF15 14/22 tissues negative, CCHF16 16/22 and CCHF19 13/22) (Fig. 3c and d). However, as a group compared to sham-vaccinated animals, we only measured significantly less viral RNA in the inguinal lymph node (Fig. 3c). As we have previously shown that humoral immunity is the primary correlate of protection^5,22,23^, we hypothesized that antibody titer would correlate with viral loads. Indeed, we found that CCHFV-specific antibody titer significantly and inversely correlated with viral load in multiple tissues, including the heart, liver, kidney, and adrenal gland (Fig. 3e). When we segregated animals into a high titer group (*n* = 4) and a low titer group (*n* = 2), compared to sham-vaccinated animals, significantly reduced viral RNA was measured in most tissues in the high-titer group but not low-titer group (Supplemental Fig. 1) further supporting the correlation between control of viral replication with levels of CCHFV-specific IgG.

**Fig. 4:**
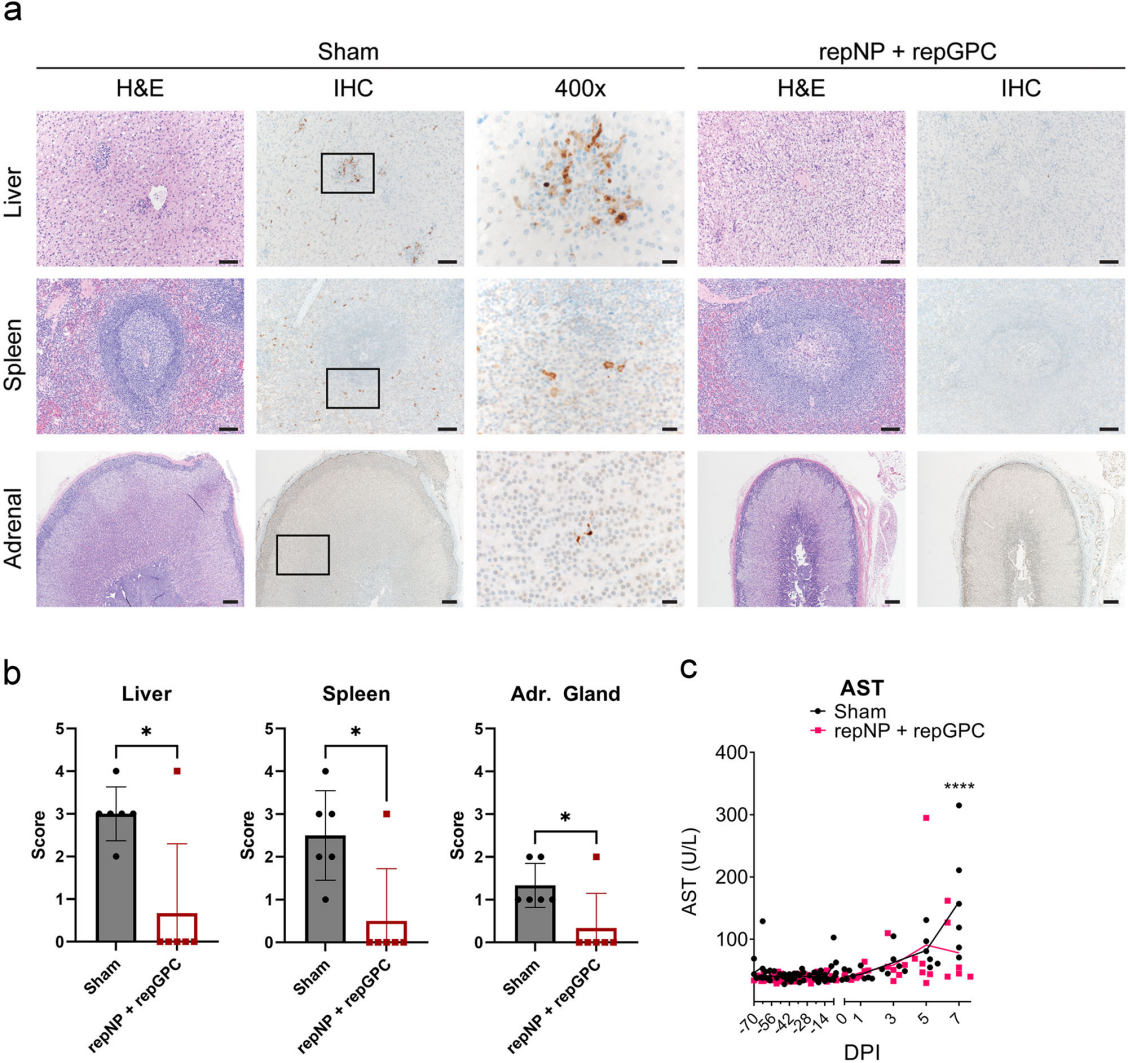
Vaccination protects against pathology and viral replication in the liver and spleen. **a** Representative image for H&E and IHC for CCHFV NP antigen in the liver, spleen, and adrenal gland of sham and repNP + repGPC animals is shown. Images are from CCHF11 for sham (liver, spleen, adrenal), CCHF17 (liver and spleen), and CCHF16 (adrenal) for repNP + repGPC vaccinated. Images are shown at ×100 for the liver and spleen, ×40 for the adrenal gland, or ×400. Scale bars indicate 200 μm (×40), 100 μm (×100), or 20 μm (×400). **b** Scores for the presence of antigen as measured by IHC are shown. 0 = none, 1 = rare/few, 2 = scattered, 3 = moderate, 4 = numerous and 5 = diffuse. **c** Serum aspartate aminotransferase (AST) levels were quantified. *P* values were calculated with Welch’s *t*-test (**b**) or two-way ANOVA with Sidak’s multiple comparisons test (**c**). *P < 0.05, ****P < 0.0001. **b** Bar shows the mean with the error bar representing the standard deviation.

**Fig. 5:**
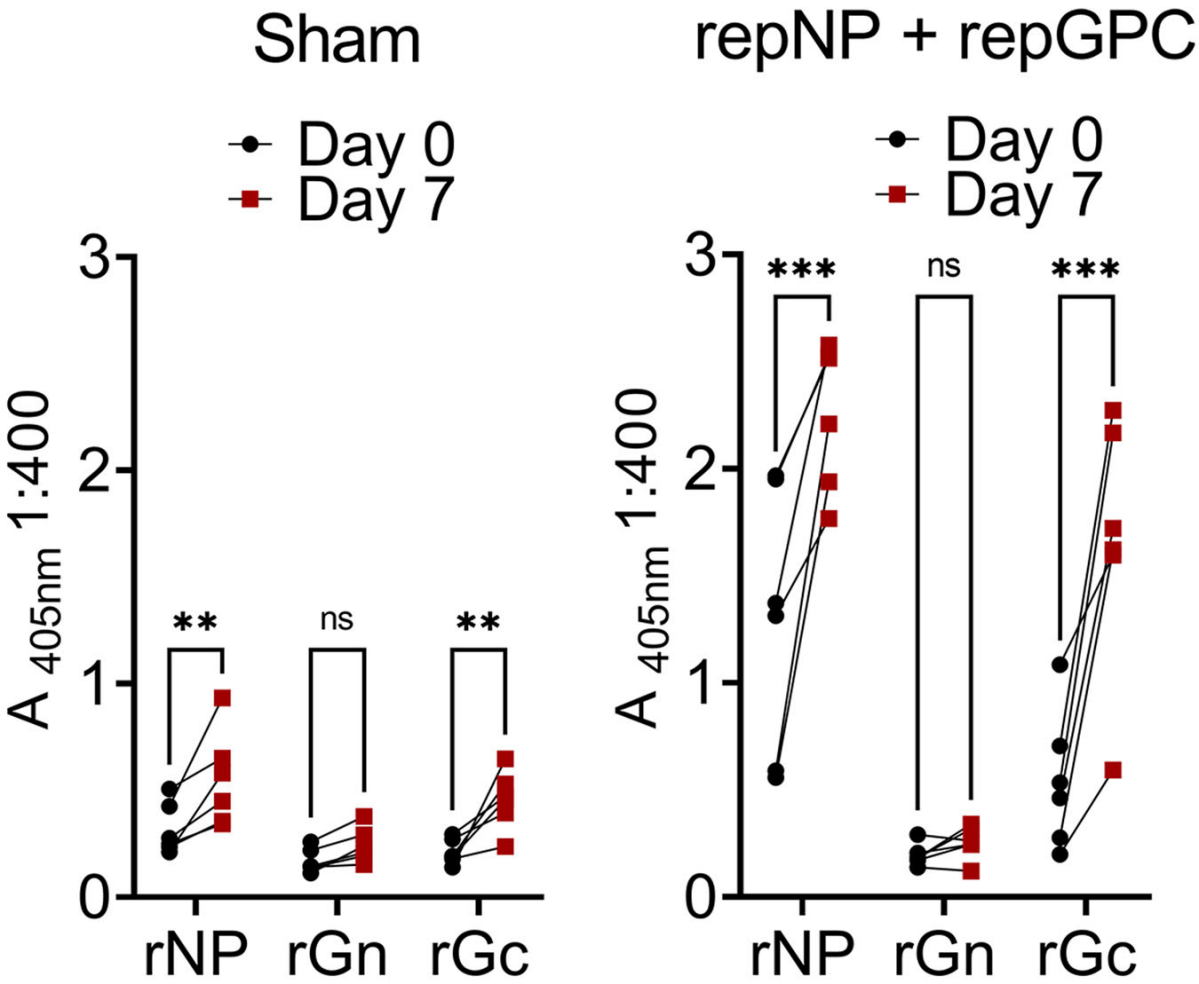
Anamnestic antibody responses in CCHFV-infected macaques. Antibody responses to indicated antigens were measured at day 0 and 7 relative to CCHFV-challenge. *P* values were calculated using two-way ANOVA with Sidak’s multiple comparisons test. **P < 0.01, ***P < 0.001.

To further evaluate viral control, we also evaluated tissue pathology and the presence of viral antigens from tissues collected on day 7 PI. Classic lesions of CCHF were observed in the liver of sham-vaccinated macaques consisting of minimal to mild hepatocellular necrosis and 5 of 6 animals had moderate numbers of lymphohistocytic nodules (Fig. 4a). In contrast, no evidence of hepatocellular necrosis was evident in the repNP + repGPC vaccinated animals and 4 of 6 had minimal numbers of lymphohistocytic nodules (Fig. 4a). No pathology was evident in the spleens of either group. In sham vaccinated macaques, immunoreactivity for the CCHFV NP was noted in hepatocytes and Kupffer cells of the liver, macrophages in splenic lymphoid follicles, and endocrine cells of the adrenal cortex and medulla (Fig. 4a and b). Immunoreactivity in the livers, spleens, and adrenal glands of vaccinated animals was significantly lower than in the sham vaccinated group (Fig. 4a and b), with only CCHF20 having detectable antigen in these tissues (Fig. 4b and Supplemental Fig. 2). Consistent with minimal pathology in the livers of repNP + repGPC vaccinated animals at necropsy, levels of aspartate aminotransferase (AST) were significantly lower in the vaccinated group at day 7 PI (Fig. 4c) although even in the sham-vaccinated group, AST levels were modest, consistent with mild-disease in this model. The complete hematological and blood chemistry parameters are provided in Supplemental Table 2, and the complete histological findings are provided in Supplemental Tables 3 and 4. We also measured anamnestic immune responses to recombinant antigens on day 7 PI. Sham and repNP + repGPC vaccinated animals had significant increases in antibody responses against NP and Gc, although the magnitude of the anamnestic response was greater in repNP + repGPC vaccinated animals (Fig. 5). The increase in Gc-specific IgG in repNP + repGPC vaccinated animals after infection suggest that repGPC vaccination primed low-levels of Gc-specific B-cells that were rapidly boosted upon infection. Together, these data suggest that the repRNA vaccine can elicit protective immunity against CCHFV and that protection correlates with CCHFV-specific antibody responses.

## Discussion

Together, our data establish an RM model of CCHF and demonstrate the protective efficacy of a repRNA vaccine for CCHFV in RM. The RM model of CCHF provides a viable alternative model to CM, exhibiting mild-to-moderate disease with similar signs of disease as observed in infected CM and mild cases of CCHF in humans. It remains unclear why the severe, terminal disease seen in the initial report of CM infected with CCHFV^15^ has not been repeated in subsequent studies by our group and others. Our serially passaged CMP-CCHFV acquired several mutations, mutations in proteins that were also mutated during serial passage in mice^24^, suggesting these proteins may have a common function in mammalian virulence. No common mutations were identified in mouse-adapted CCHFV and CMP-CCHFV. However, unlike the mouse-adapted strain of CCHFV, disease upon infection of RM with CMP-CCHFV remains mild, suggesting additional host or viral barriers to severe disease exist in NHPs.

Our data and previous data from mice and NHPs by our group and others^5,16,18,25,26^ add to a growing body of evidence that vaccine-expressed CCHFV NP can confer remarkable protection against CCHFV, likely through protective non-neutralizing antibodies. In our study, CCHFV-specific antibodies significantly and inversely correlated with viral loads in multiple tissues, including key tissues such as the liver, kidney, heart, and lung tissue. Further, our recombinant antigen ELISAs indicated humoral immunity was mainly directed against NP with little humoral or cellular immunity directed against GPC prior to challenge. It is unclear why some animals developed higher responses to CCHFV than others, nor is it clear why CCHF20 had a striking drop in titers at the time of challenge. This could be due to the outbred nature of the animals. Nevertheless, all animals developed a CCHFV-specific response following vaccination, demonstrating that our repRNA platform is immunogenic in RM.

To date, only a DNA-based vaccine has been evaluated in NHPs for CCHFV^16,18^. In that approach, prime-boost-boost or prime-boost with DNA plasmids expressing the CCHFV NP or GPC conferred significant protection against multiple parameters of disease in CCHFV-infected CM^16,18^. Similar to our study here, NP-specific antibodies appear to be the major correlate of protection for the DNA vaccine against CCHFV in the CM model^16,18^, and CM vaccinated with DNA-expressed NP alone were protected from CCHFV challenge^18^. Our repRNA vaccine may provide quicker immunity than the DNA-based approach. CCHFV-specific immune responses after prime-only vaccination with the DNA vaccine were undetectable^16,18^, suggesting multiple immunizations will be required for protection. In contrast, we observed CCHFV-specific humoral immunity after a single immunization, suggesting this repRNA platform may provide protection after a single immunization. However, our data also suggest that higher CCHFV-specific antibody responses confer greater protection, and thus, boosting may still be warranted to confer optimal immunity with our vaccine.

Although we saw enhanced protection when mice were vaccinated with repNP and repGPC compared to repNP alone^5^, it is unclear if the inclusion of the repGPC component in this study contributed to protection. Limited GPC-specific immunity was observed prior to the challenge, suggesting the repGPC component was largely non-immunogenic. In mice, the repGPC elicited potent T-cell immunity but little-to-no antibody either before or after challenge^5,23^. After the challenge, vaccinated RM had a significant anamnestic response to the Gc antigen and thus, in RM, repGPC may have primed a low amount of immunity that was rapidly boosted upon challenge. Thus, it is possible that this rapid anamnestic immunity to the Gc antigen may contribute to protection. Ongoing studies are evaluating the contribution of the individual antigens in protection in NHP models of CCHF. We also cannot exclude the possibility that antibodies elicited by our vaccine expressing the strain Hoti GPC had impaired binding to our recombinant antigens based on strain 10200.

Our data also add to the complexities of vaccine antigens for CCHFV. Similar to our findings here, CM vaccinated with just a DNA-based vaccine expressing the GPC failed to develop significant antibody responses against CCHFV^16,18^, suggesting that the failure to induce GPC-directed antibodies by our vaccine prior to the challenge is not unique to this platform. However, CM vaccinated with our DNA-based vaccine developed significant cellular immunity against the GPC prior to challenge^16,18^. It is unclear why the repGPC vaccine failed to elicit significant immunity in RM. The DNA-expressed GPC was fused to a ubiquitin tag^16^, which may have promoted the degradation and presentation of peptides to T-cells. The CCHFV GPC is more complex than most other bunyaviruses, encoding multiple accessory proteins and undergoing several proteolytic processing events required for proper virion formation^27,28^. We cannot exclude the possibility that GPC expressed in the context of an alphavirus replicon may be improperly expressed and presented, leading to poor immunogenicity. Lastly, the accessory proteins in the GPC have unclear function^2^. The CCHFV GPC encodes a mucin-like domain^29^, and a similar domain in Ebola virus GP has been shown to shield MHC-I on infected cells from CD8 T-cells^30^. It is possible that the accessory proteins within the CCHFV GPC may modulate host immunity to epitopes found within the GPC.

Our data here and our previous data evaluating a DNA vaccine in CM^16,18^ contrast with several other vaccine platforms that have reported significant anti-GPC antibodies, including neutralizing antibodies, in mouse models^31–34^. However, antibody responses may be dispensable for GPC vaccine-mediated protection. A DNA-based vaccine expressing only the GPC elicited significant humoral immunity but required CD8 T-cells, not antibodies, to confer protection in mice^35^. Further, we have found that CCHFV infection of naïve, wild-type C57BL6/J mice resulted in >75% of T-cells responding to a single peptide in Gc^36^, a level of immunodominance not seen in humans^37^ or vaccinated CM^16^. These contrasting findings across vaccine platforms and animal models, including our own showing strong cellular immunity elicited by our repGPC in mice^5,23^ but not RM, suggest that immunogenicity and efficacy measured in inbred mouse models may not always translate to higher organisms such as NHPs or humans. We have also previously shown the repRNA platform to induce significant cellular immunity against SARS-CoV-2 in NHP models^13,14^ demonstrating that this vaccine platform can induce cellular immunity in NHPs. Thus, the failure to elicit cellular immunity here may be antigen-specific. Cumulatively, the varying data for immune responses elicited by vaccines against CCHFV seen across multiple studies, platforms, and animal models suggest the vaccine platform and model utilized are critical determinants in host responses and the protective capacity of vaccine-expressed CCHFV antigens. Thus, it will be critical to mechanistically identify the specific immune responses that confer protection against CCHFV for each vaccine and, as vaccine candidates move into human clinical trials, which models most accurately predict vaccine efficacy in humans.

Our study has some important limitations. First, CMP-CCHFV infection of RM results in mild disease, and thus, we were unable to evaluate whether repRNA vaccination can protect against terminal disease. Nevertheless, this limitation is present for both the RM and CM models. Further, asymptomatic and subclinical infections may represent a substantial fraction, if not the majority, of CCHFV infections in humans^1^, and thus, this limitation may reflect the accuracy with which CM and RM model human CCHFV infection. Beyond protection from disease, our data support the use of RM and CM for pre-clinical immunogenicity studies in an outbred population to support advancement of candidate vaccines to human clinical trials. Second, the variable immune response to vaccination observed in our study cohort suggests that continued optimization of the vaccine platform is necessary to drive greater immune responses to vaccine-encoded antigens in genetically variable populations. The repNP used here encoded a V5-epitope tag as in our initial mouse studies^5^, and we have recently shown that removal of this tag may lead to increased immunogenicity^23^. Ongoing studies in NHPs are evaluating a repNP with this tag removed and refined GPC antigens. Third, our vaccine encodes antigens based on CCHFV strain Hoti and although CMP-CCHFV accumulated several mutations, this still represents a largely homologous challenge. CCHFV has substantial genetic diversity, differing by >5% in the NP and over 25% in the GPC^38^, and thus, an effective vaccine will need to protect against diverse strains of CCHFV. We have shown in mice that our repNP vaccine and passive transfer of NP-immune serum can protect against a highly divergent strain of CCHFV^5,22,23^, making the more conserved NP a promising vaccine-encoded antigen. Fourthly, we did not evaluate the durability of immune responses to CCHFV following repRNA vaccination. Given the continued circulation of CCHFV within endemic areas that have limited healthcare resources, an optimal vaccine for CCHFV will induce durable immune responses and avoid the need for repeat vaccinations. However, we have shown that this vaccine platform could induce long-term protective immunity against SARS-CoV-2 in NHPs^13^. Lastly, we cannot rule out the role of nonspecific, antigen-independent immune responses in protection from CCHFV infection in RMs due to the 2-fold dose difference between the negative control and experimental groups. However, given our previous data demonstrating minimal magnitude and duration of systemic innate immune responses to repRNA/LION^8^ as well as time-limited antigen production following intramuscular administration^39^, and significant correlation between CCHFV-specific IgG and reduced viral loads, we perceive the risk of such confounding effects to be minimal as animals were challenged 4 weeks after the last immunization.

Cumulatively, our data establishes an RM model of CCHF, providing an additional NHP model for CCHF. Disease in RM presents similarly to the mild disease reported in CM and in humans but nevertheless enables evaluation of candidate vaccines and therapeutics against viral replication, tissue pathology, and hematological disturbances. Our data also demonstrate the immunogenicity and protective efficacy of our repRNA vaccine for CCHFV in an NHP model, add to our understanding of the correlates of protection for CCHFV, and demonstrate that protection correlated with CCHFV-specific humoral immunity. Our data also support the hypothesis that antibody against NP is the primary correlate of protection with our vaccine. Cumulatively, our data support the continued development of this vaccine for CCHFV.

## Methods

### Animals, biosafety and ethics

All infectious work with CCHFV and sample inactivation was performed in the maximum containment laboratory in accordance with standard operating procedures approved by the Rocky Mountain Laboratories Institutional Biosafety Committee, Division of Intramural Research, National Institute of Allergy and Infectious Diseases, National Institutes of Health (Hamilton, MT, USA). All animal work was performed in strict accordance with the recommendations described in the Guide for the Care and Use of Laboratory Animals of the Office of Animal Welfare, National Institutes of Health, and the Animal Welfare Act of the US Department of Agriculture, in an AAALACi-accredited facility. Indian-origin RM was individually housed in adjoining primate cages that enabled social interaction under controlled conditions of humidity, temperature, and light (12-h light/12-h dark cycles). Water was available ad libitum. Animals were monitored at least twice daily (pre- and post-infection) and fed commercial monkey chow, treats, and fruit twice a day by trained personnel. Environmental enrichment consisted of human interaction, manipulanda, visual enrichment and audio enrichment. All procedures on nonhuman primates were performed by board-certified clinical veterinarians who also provided veterinary oversight of the study. All procedures were done on anesthetized animals. Anesthesia was performed by intramuscular injection of Ketamine/HCL (10 mg/kg) or Telazol (3–3.5 mg/kg), and anesthesia was maintained as necessary with isoflurane (1–5%, inhalation). Animals were euthanized under deep anesthesia (5 mg/kg Telazol) and intracardiac administration of 1 mL/5 kg euthanasia solution. All necropsies were performed by board-certified veterinary pathologists. Blood chemistry and hematology were assessed using a Vetscan2 with Preventive Care profile disks (Abaxis, USA) and ProCyte DX (IDEXX Labs, USA), respectively. Study 1 used eight Indian origin, male and female RM between the ages of 2.9 and 8.6 years. Our vaccination study used Indian origin, male and female RM between the ages of 2.1 and 12.2 years of age. The origin, age, sex, and complete blood chemistry and hematology data for each study are provided in Supplemental Table 1.

### Vaccine and vaccinations

In our RM studies, the repNP and repGPC expressed the NP and GPC of CCHFV strain Hoti as previously described^5^. RNA was delivered by complexing to LION as previously described^14^. Vaccination was performed by a single intramuscular injection consisting of 25 μg of each RNA. This resulted in repNP + repGPC animals receiving 50 μg of RNA while sham animals received 25 μg of RNA. Six-weeks after prime-vaccination animals were boosted with identical vaccinations. Vaccination appeared well tolerated with no adverse events observed following vaccinations.

### Virus challenge

Animals were challenged with 1 × 10^5^ TCID_50_ of CCHFV CMP-CCHFV divided between subcutaneous injections over the dorsal thorax and intravenously through the saphenous vein as previously described^15^. Our challenge stock of CMP-CCHFV Hoti was propagated and tittered on SW-13 cells and sequenced as previously described for CCHFV strain Hoti^15,40^ .

### ELISA

Antibody to gamma-irradiated whole-virus antigen from CCHFV Hoti infected cells was measured as previously described^15^. Recombinant antigen ELISA was performed as above but using specified antigens (Native Antigen Company) coated on Maxisorp plates (Nunc) at 100 ng/well in PBS. Endpoint titers were calculated using a cutoff defined as the average of sham vaccinated animals at day 0 PI at the 1:400 dilution + (3*standard deviations). The dilution at the cutoff was interpolated using a sigmoidal 4PL fit in Prism.

### IFNγ ELISpot

Cryopreserved peripheral blood mononuclear cells (PBMCs) were collected on some exams and evaluated for IFNγ production in response to overlapping peptides spanning the entire CCHFV NP or GPC using a 384-well human IFNγ ELISpot kit (Immunospot) as previously described^18^. All measurements were performed in duplicate. The number of spots in cells stimulated with the DMSO vehicle was subtracted from cells stimulated with CCHFV peptides or PMA/Ionomycin and counts normalized to 1 × 10^6^ cells.

### RT-qPCR

Viral RNA in blood and tissues was quantified in RNA extracted using RNeasy and Qiamp Viral RNA kits (Qiagen) according to the manufacturer’s instructions. RT-qPCR and quantification by standard curve were as previously described^24^. The limit of quantitation (LoQ) was defined as the copy # of the last standard to amplify, while the limit of detection (LoD) was defined as the value given by a Ct value of 40.

### Histology and IHC

Histology and immunohistochemistry for the CCHFV NP antigen were performed on formalin-fixed tissue sections as previously described^24^. Tissue sections were evaluated by board-certified pathologists who were blinded to study groups. H&E sections were scored according to 0 = No lesions, 1 = minimal (1–10%), 2 = mild (11–25%), 3 = moderate (26–50%), 4 = marked (51–75%) and 5 = severe (76–100%). IHC was scored according to 0 = none, 1 = rare/few, 2 = scattered, 3 = moderate, 4 = numerous and 5 = diffuse.

### Statistics

Indicated statistical tests were performed using Prism 9 (GraphPad). Spearman correlation analyses between log-transformed IgG endpoint titer and viral loads were performed using the JMP® statistical analysis software. Asterisks indicate correlations that were statistically significant (*p*-value < 0.05). The *X*–*Y* scatterplots show 95% confidence density ellipses for normally distributed data.

### Reporting summary

Further information on research design is available in the Nature Research Reporting Summary linked to this article.

### Supplementary information

Supplemental Figures and Tables

Reporting summary

## Data Availability

All data underlying the figures is available upon reasonable request.
