# Supplementary material for: A replicating RNA vaccine confers protection in a rhesus macaque model of Crimean-Congo hemorrhagic fever
Source: NPJ Vaccines. 2024 May 20;9:86. doi: 10.1038/s41541-024-00887-z (PMC11106275; doi:10.1038/s41541-024-00887-z)
Supplement: Supplementary file 1 — Supplemental Figures and Tables [file 41541_2024_887_MOESM1_ESM.pdf]

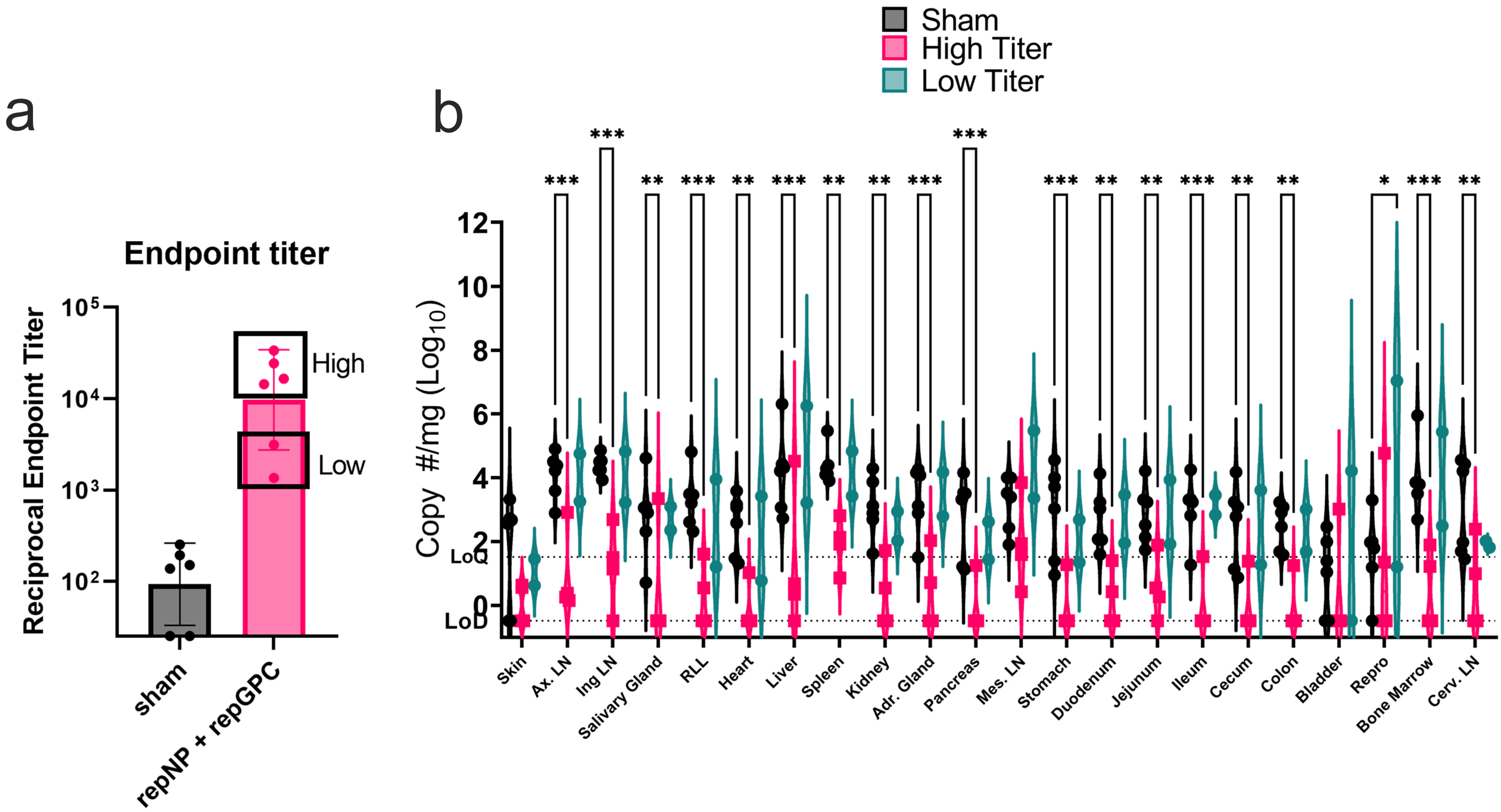

Supplemental Figure 1: Control of viral RNA in tissues. CM were segregated into a high (n = 4) and low (n = 2) titer group based on endpoint titers at day 0 PI (a). On day 7 PI, viral RNA in indicated tissues was quantified by qRT-PCR (b). Statistical comparisons were performed between high or low titer and sham vaccinated animals. P values calculated with two-way ANOVA with Dunett's multiple comparison test. * $P < 0.05$, ** $P < 0.01$, *** $P < 0.001$. (a) Bar represents mean with error bars representing standard deviation. (b) Data is shown as violin plot.

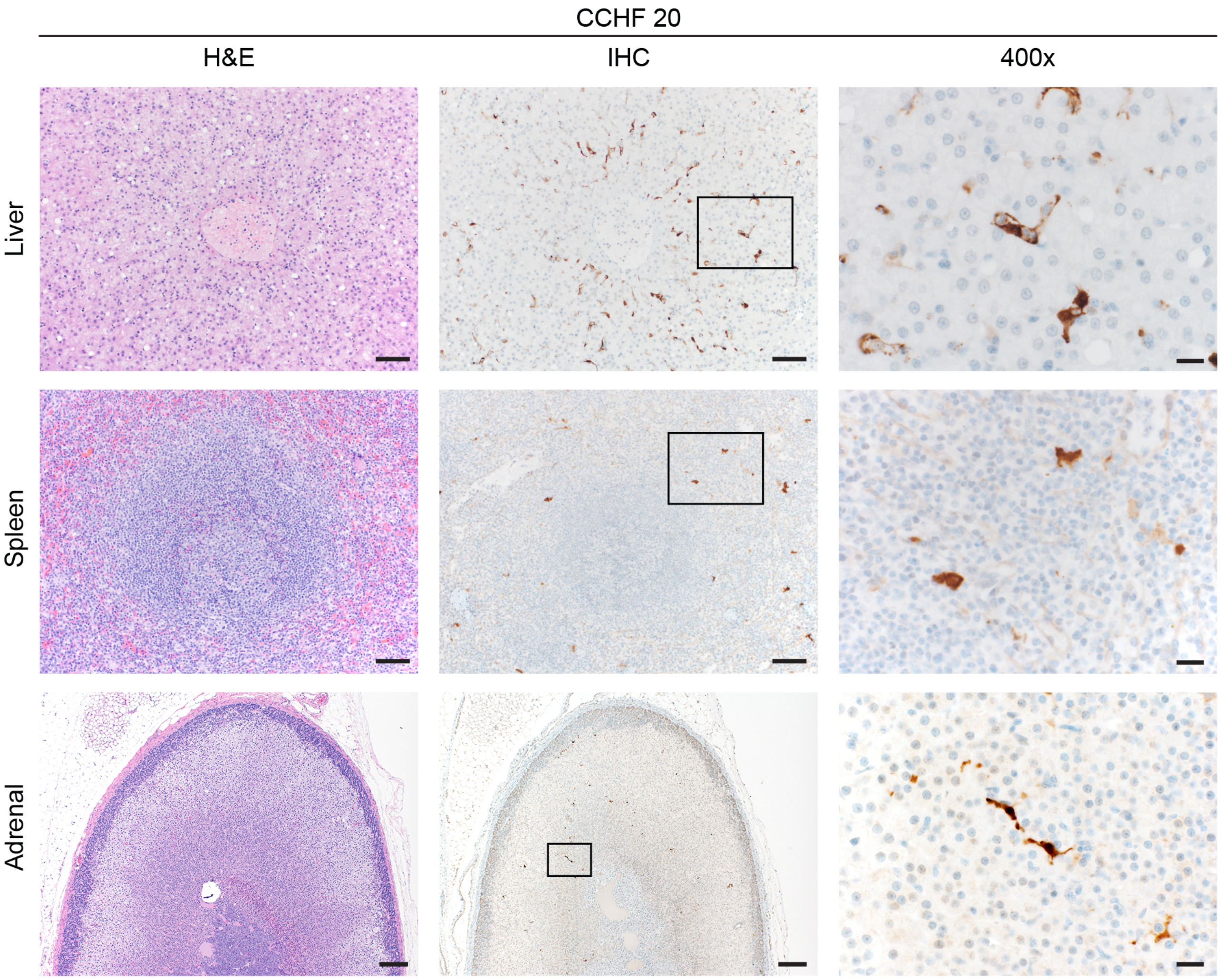

Supplemental Figure 2: H&E and IHC sections of CCHF20. Images are shown at 100x for liver and spleen, 40x for adrenal gland or 400x. Scale bars indicate 200 µm (40x), 100µm (100x) or 20µm (400x).

Supplemental Table 1: Complete Blood Chemistry and Hematology for Study 1

Indian Origin

Study ID	Sex	Age (yrs)
CCHF01	M	2.9
CCHF02	M	3.6
CCHF03	F	3.7
CCHF04	F	3.9
CCHF05	M	7.9
CCHF06	M	8.6
CCHF07	F	5.6
CCHF08	F	7.6

Study Day	Animal ID	BUN (mg/dL)	Creatinine (mg/dL)	ALT (U/L)	ALP (U/L)	AST (U/L)	T Bilirubin (mg/dL)	Glucose (mg/dL)	Calcium (mg/dL)	T Protein (g/dL)	Albumin (g/dL)	Globulin (g/dL)	Na+ (mmol/L)	K+ (mmol/L)	CL- (mmol/L)	tC02 (mmol/L)	Hem	Lip	Ict
D0	CCHF01	11	0.6	36	687	37	0.3	69	10.5	6.5	4.8	1.7	152	3.9	116	19	0	0	0
D1	CCHF01	11	0.8	33	637	42	0.3	86	10.8	6.5	4.7	1.7	152	4.1	112	23	0	0	0
D3	CCHF01	13	0.8	34	646	35	0.3	75	10.4	6.7	4.8	1.9	154	3.9	111	24	0	0	0
D5	CCHF01	11	0.7	36	624	39	0.3	75	10.3	6.9	4.9	2.0	156	4.1	117	24	0	0	0
D7	CCHF01	11	1.0	45	531	60	0.3	76	10.1	6.2	4.5	1.7	151	3.5	114	23	0	0	0
D9	CCHF01	9	0.5	35	490	40	0.3	75	10.4	6.4	4.6	1.8	150	4.0	115	24	0	0	0
D14	CCHF01	8	0.7	39	586	34	0.3	73	10.6	6.7	4.8	1.9	153	3.8	115	23	0	0	0
D28	CCHF01	11	0.7	48	612	42	0.2	75	10.5	6.4	4.8	1.6	149	4.0	124	29	0	0	0
D0	CCHF02	13	1.2	15	311	29	0.2	78	10.6	7.2	5.4	1.8	151	4.5	114	19	0	0	0
D1	CCHF02	12	1.0	21	297	32	0.3	92	10.7	7.0	5.3	1.8	152	4.3	112	21	0	0	0
D3	CCHF02	14	1.0	20	319	34	0.3	93	10.4	7.3	5.2	2.1	149	4.7	108	22	0	0	0
D5	CCHF02	14	0.8	27	291	39	0.3	82	9.6	6.9	5.1	1.9	147	4.3	113	26	0	0	0
D7	CCHF02	15	1.3	49	256	150	0.2	113	9.2	6.6	4.7	1.9	146	3.7	109	22	0	0	0
D9	CCHF02	11	0.9	64	211	74	0.2	83	9.5	6.5	4.6	1.9	149	4.7	114	26	0	0	0
D14	CCHF02	11	0.8	23	227	31	0.2	83	10.2	7.0	5.0	2.0	149	4.6	113	27	0	0	0
D28	CCHF02	13	0.9	19	272	31	0.3	71	11.0	7.2	5.4	1.8	149	4.4	113	29	0	0	0
D0	CCHF03	13	0.8	16	281	24	0.2	76	10.4	6.6	4.8	1.7	147	4.5	114	20	0	0	0
D1	CCHF03	12	1.1	14	277	24	0.3	94	10.4	6.3	4.7	1.7	145	4.5	115	22	0	0	0
D3	CCHF03	14	0.9	25	299	33	0.3	80	9.9	6.3	4.5	1.8	143	4.7	112	20	0	0	0
D5	CCHF03	11	1.0	30	280	34	0.3	82	9.8	6.1	4.3	1.8	145	4.5	116	17	0	0	0
D7	CCHF03	14	0.8	45	329	121	0.4	87	9.7	6.6	4.4	2.2	147	4.0	118	16	0	0	0
D9	CCHF03	12	0.7	39	293	42	0.3	104	10.1	6.7	4.4	2.3	146	4.3	114	19	0	0	0
D14	CCHF03	14	0.6	20	367	29	0.2	78	11.0	7.1	4.7	2.4	146	5.0	109	25	0	0	0
D28	CCHF03	13	1.1	14	272	21	0.3	69	10.4	6.3	4.5	1.8	143	4.5	115	22	0	0	0
D0	CCHF04	11	0.9	27	170	27	0.3	98	11.0	7.3	4.8	2.5	148	4.7	115	25	0	0	0
D1	CCHF04	9	0.8	28	170	32	0.3	126	10.9	7.0	4.7	2.3	149	4.2	112	25	0	0	0
D3	CCHF04	11	0.8	34	164	42	0.4	85	10.1	6.5	4.3	2.2	146	4.1	113	25	0	0	0
D5	CCHF04	10	1.0	43	181	73	0.4	99	9.7	6.4	4.2	2.2	144	4.6	112	24	0	0	0
D7	CCHF04	10	1.0	48	150	75	0.4	111	9.5	6.5	4.1	2.3	145	4.3	113	22	0	0	0
D9	CCHF04	12	0.7	36	128	38	0.4	120	9.8	6.5	4.2	2.3	147	4.2	116	25	0	0	0
D14	CCHF04	10	0.7	20	161	26	0.3	92	10.4	7.3	4.6	2.7	148	4.0	119	28	0	0	0
D28	CCHF04	10	1.0	22	195	24	0.3	124	10.9	7.3	4.9	2.4	149	4.9	118	29	0	0	0
D0	CCHF05	17	1.1	67	126	34	0.3	55	10.4	6.7	4.9	1.8	148	4.4	120	25	1	0	0
D1	CCHF05	18	1.0	72	110	41	0.4	85	10.2	6.6	4.7	1.9	147	4.3	110	28	0	0	0
D3	CCHF05	14	1.3	62	114	54	0.5	71	10.3	6.8	4.6	2.2	144	4.3	114	25	0	0	0
D5	CCHF05	12	1.1	63	109	89	0.5	92	9.5	6.5	4.4	2.0	143	4.2	119	26	0	0	0
D7	CCHF05	15	1.5	238	128	785	0.4	79	9.6	6.5	4.1	2.4	145	4.0	114	24	0	1	0
D9	CCHF05	16	0.9	152	106	240	0.3	82	9.1	5.9	3.7	2.1	140	4.3	108	27	0	0	0
D14	CCHF05	14	0.7	70	118	54	0.3	71	10.2	6.6	4.2	2.4	147	4.3	114	29	0	0	0
D0	CCHF06	11	1.4	28	101	26	0.4	77	9.7	6.6	4.9	1.6	151	4.3	115	26	0	0	0
D1	CCHF06	12	1.5	30	100	33	0.4	85	9.7	6.3	4.7	1.6	147	4.2	113	27	0	0	0
D3	CCHF06	9	1.1	36	103	44	0.4	74	9.5	6.3	4.5	1.7	150	4.3	116	27	1	0	0
D5	CCHF06	9	1.1	44	87	50	0.4	76	9.5	6.4	4.6	1.8	148	4.2	116	30	0	0	0
D7	CCHF06	11	0.9	59	95	80	0.3	78	9.4	6.4	4.5	1.9	148	4.1	116	26	0	0	0
D9	CCHF06	9	1.3	43	95	36	0.4	77	9.5	6.7	4.6	2.1	146	4.2	115	28	0	0	0
D14	CCHF06	11	0.9	31	102	27	0.4	76	9.9	6.7	4.8	2.0	148	4.1	118	27	0	0	0
D28	CCHF06	11	1.1	28	104	27	0.4	75	9.8	6.9	4.9	1.9	147	4.4	118	29	0	0	0
D0	CCHF07	11	1.2	37	125	24	0.3	82	11.0	7.5	5.0	2.4	149	4.7	113	28	0	0	0
D1	CCHF07	11	1.2	29	104	38	0.4	83	10.4	7.0	4.7	2.4	147	4.3	107	28	0	0	0
D3	CCHF07	17	1.0	85	127	154	0.5	72	9.7	6.6	4.3	2.3	145	3.9	113	24	0	0	0
D5	CCHF07	14	1.3	454	159	497	0.4	84	9.4	6.1	4.1	2.0	145	4.1	113	27	0	0	0
D7	CCHF07	13	0.7	742	200	401	0.7	79	9.5	6.1	3.9	2.2	148	3.9	112	28	0	0	0
D9	CCHF07	11	1.2	288	171	68	0.4	80	9.7	6.7	4.0	2.7	149	4.2	115	30	0	0	0
D14	CCHF07	8	0.5	69	165	30	0.4	75	10.7	7.1	4.5	2.6	151	4.6	112	29	0	0	0
D0	CCHF08	10	1.0	87	128	33	0.3	78	10.1	6.8	4.6	2.2	148	4.0	115	26	0	0	0
D1	CCHF08	9	0.6	94	159	48	0.3	87	10.0	6.3	4.3	2.0	145	3.9	109	27	0	0	0
D3	CCHF08	10	0.5	83	148	49	0.3	68	10.1	6.7	4.4	2.3	150	4.2	115	27	0	0	0
D5	CCHF08	7	0.6	121	186	57	0.3	75	9.3	6.3	4.3	2.0	143	3.5	115	27	0	0	0
D7	CCHF08	10	0.3	112	157	87	0.4	73	9.6	6.2	4.1	2.1	141	3.2	110	27	0	0	0
D9	CCHF08	7	0.6	110	150	58	0.3	77	9.3	6.4	4.2	2.2	145	3.8	112	30	0	0	0
D14	CCHF08	10	0.8	76	146	35	0.3	79	9.6	6.6	4.3	2.3	147	3.9	115	28	0	0	0
D28	CCHF08	10	0.6	112	114	47	0.3	62	9.9	6.4	4.3	2.0	146	3.8	119	28	0	0	0

Study Day	Animal ID	RBC (M/uL)	WBC (K/uL)	HGB (g/dL)	NEUT# (K/uL)	NEUT% (%)	HCT (%)	LYMPH# (K/uL)	LYMPH% (%)	MCV (fL)	MONO# (K/uL)	MONO% (%)	MCH (pg)	EOS# (K/uL)	EOS% (%)	MCHC (g/dL)	BASO# (K/uL)	BASO% (%)	RDW SD (fL)	RDW CV (%)	PLT (K/uL)	MPV (fL)	RET# (K/uL)	RET% (%)
D0	CCHF01	6.25	6.58	13	2.97	45.2	38.2	2.85	43.3	61.1	0.64	9.7	20.8	0.12	1.8	34	0	0	33.1	16.2	336	9.8	51.3	0.82
D1	CCHF01	5.62	6.81	11.7	2.2	32.4	35.9	3.95	58	63.9	0.52	7.6	20.8	0.13	1.9	32.6	0.01	0.1	35.1	16.2	341	9.9	62.9	1.12
D3	CCHF01	5.8	5.73	12.2	1.03	18	37.2	3.99	69.6	64.1	0.71	12.4	21	0	0	32.8	0	0	34.8	15.9	370	9.4	61.5	1.06
D5	CCHF01	5.49	4.4	11.4	0.94	21.4	35.3	2.83	64.3	64.3	0.58	13.2	20.8	0.05	1.1	32.3	0	0	35	16	341	9.6	66.4	1.21
D7	CCHF01	5.41	6.25	11.3	0.93	14.9	34.8	4.61	73.8	64.3	0.69	11	20.9	0.02	0.3	32.5	0	0	35.1	16	347	9.7	54.6	1.01
D9	CCHF01	5.28	9.97	11	0.95	9.5	34.2	7.31	73.3	64.8	1.71	17.2	20.8	0	0	32.2	0	0	34.5	15.6	406	9.7	69.2	1.31
D14	CCHF01	5.86	9.88	12.4	1.59	16.1	37.8	6.27	63.5	64.5	1.9	19.2	21.2	0.12	1.2	32.8	0	0	34.8	16.2	381	9.3	117.2	2
D28	CCHF01	5.95	8.3	12.7	2.66	32	39.1	4.87	58.7	65.7	0.77	9.3	21.3	0	0	32.5	0	0	35.7	16	378	9.8	73.2	1.23
D0	CCHF02	6.55	10.91	14.7	8.24	75.6	42.5	2.22	20.3	64.9	0.44	4	22.4	0	0	34.6	0.01	0.1	37.2	18.3	421	11	45.9	0.7
D1	CCHF02	5.99	12.13	13.2	9.57	79	40.3	1.86	15.3	67.3	0.5	4.1	22	0	0	32.8	0.2	1.6	38.8	17.1	378	11.3	39.5	0.66
D3	CCHF02	6.11	6.66	13.6	4.36	65.4	41.1	2.07	31.1	67.3	0.22	3.3	22.3	0.01	0.2	33.1	0	0	38.9	17.4	379	11.5	30.6	0.5
D5	CCHF02	5.69	2.51	12.8	1.42	56.5	38.3	0.83	33.1	67.3	0.26	10.4	22.5	0	0	33.4	0	0	38.2	16.5	299	11.1	15.4	0.27
D7	CCHF02	5.96	3.95	13.3	0.94	23.8	39.3	2.18	55.2	65.9	0.83	21	22.3	0	0	33.8	0	0	36.3	16.3	119	15.7	20.3	0.34
D7 (rerun)	CCHF02	5.99	4.17	13.3	1.08	25.9	39.5	2.74	65.7	65.9	0.35	8.4	22.2	0	0	33.7	0	0	36.5	16.1	139	11.9	22.2	0.37
D9	CCHF02	5.7	9.84	12.6	2.15	21.9	37.6	6.93	70.4	66	0.76	7.7	22.1	0	0	33.5	0	0	36.2	15.8	211	11.1	17.1	0.3
D14	CCHF02	5.98	10.15	13.3	5.21	51.3	39.6	3.49	34.4	66.2	1.45	14.3	22.2	0	0	33.6	0	0	38.5	17.9	408	11.3	108.2	1.81
D28	CCHF02	6.42	9.77	14.9	5.89	60.3	43.8	3.27	33.5	68.2	0.58	5.9	23.2	0.02	0.2	34	0.01	0.1	36.8	16.1	332	11.6	62.3	0.97
D0	CCHF03	6.19	10.37	14.1	7.04	67.9	40.4	2.6	25.1	65.3	0.72	6.9	22.8	0.01	0.1	34.9	0	0	33	14.8	340	10.5	105.8	1.71
D1	CCHF03	5.75	19.39	12.8	16.52	85.1	38.5	2.28	11.8	67	0.54	2.8	22.3	0	0	33.2	0.05	0.3	34.1	14.9	332	11.4	86.8	1.51
D3	CCHF03	5.96	6.26	13.5	3.76	60	39.2	2.2	35.1	65.8	0.26	4.2	22.7	0.03	0.5	34.4	0.01	0.2	33	14.7	311	11.4	106.1	1.78
D5	CCHF03	5.71	4.97	12.9	3.3	66.4	37.8	1.38	27.8	66.2	0.28	5.6	22.6	0.01	0.2	34.1	0	0	33.6	14.9	360	10.6	121.6	2.13
D7	CCHF03	6.12	4.67	13.8	1.4	30	40.6	2.91	62.3	66.3	0.35	7.5	22.5	0.01	0.2	34	0	0	34.1	15.1	204	11.6	146.3	2.39
D7 (rerun)	CCHF03	6.15	4.68	13.7	1.38	29.5	40.8	2.46	52.6	66.3	0.83	17.7	22.3	0.01	0.2	33.6	0	0	34.5	15.1	211	12	156.2	2.54
D9	CCHF03	6.11	15.6	13.6	6.25	40	40.9	8.38	53.7	66.9	0.95	6.1	22.3	0.01	0.1	33.3	0.01	0.1	34.9	15.8	251	12.4	180.9	2.96
D14	CCHF03	6.58	14.47	14.6	10.06	69.6	43.7	3.84	26.5	66.4	0.57	3.9	22.2	0	0	33.4	0	0	35.4	16.7	417	11.3	248.1	3.77
D28	CCHF03	6.31	16.1	14.5	11.49	71.3	42.8	3.51	21.8	67.8	1.01	6.3	23	0.08	0.5	33.9	0.01	0.1	34.9	15.2	344	10.6	136.3	2.16
D0	CCHF04	6.31	6.83	14.6	4.74	69.4	42.9	1.9	27.8	68	0.19	2.8	23.1	0	0	34	0	0	35.4	15.6	526	9.4	69.4	1.1
D1	CCHF04	5.55	5.27	12.6	3.3	62.6	38.4	1.65	31.3	69.2	0.3	5.7	22.7	0.01	0.2	32.8	0.01	0.2	35.6	15.1	484	9.8	56.1	1.01
D3	CCHF04	5.26	3.92	12.1	2.36	60.2	35.7	1.37	34.9	67.9	0.18	4.6	23	0	0	33.9	0.01	0.3	35.6	15.3	374	10.3	54.2	1.03
D5	CCHF04	5.32	3.68	11.9	2.33	63.3	36.1	1.13	30.7	67.9	0.22	6	22.4	0	0	33	0	0	35.2	15.1	372	10.4	51.1	0.96
D7	CCHF04	5.55	4.72	12.6	0.7	14.8	37.7	3.33	70.6	67.9	0.68	14.4	22.7	0	0	33.4	0.01	0.2	35	15	377	10.6	66	1.19
D9	CCHF04	5.21	14.02	11.6	3.93	28.1	35.7	9.1	64.9	68.5	0.96	6.8	22.3	0	0	32.5	0.03	0.2	36.3	15.6	339	10.2	218.3	4.19
D14	CCHF04	6.05	13.76	13.7	8.79	63.8	41.2	3.2	23.3	68.1	1.76	12.8	22.6	0	0	33.3	0.01	0.1	34.9	14.9	444	10	170	2.81
D28	CCHF04	6.28	6.59	14.1	3.49	53	43	2.34	35.5	68.5	0.76	11.5	22.5	0	0	32.8	0	0	37	16.6	413	10.2	60.3	0.96
D0	CCHF05	6.08	18.54	15.2	16.54	89.2	44.1	1.32	7.1	72.5	0.65	3.5	25	0.03	0.2	34.5	0	0	34.1	13.6	290	9.3	113.7	1.87
D1	CCHF05	5.89	7.89	14.3	5.75	72.9	43.6	1.58	20	74	0.37	4.7	24.3	0.14	1.8	32.8	0.05	0.6	34.3	13.4	293	9.5	100.7	1.71
D3	CCHF05	5.87	4.48	14.3	2.7	60.3	43.2	1.3	29	73.6	0.44	9.8	24.4	0.04	0.9	33.1	0	0	34.3	13.5	262	9.6	57.5	0.98
D5	CCHF05	5.67	2.8	13.7	1.83	65.4	41.2	0.63	22.5	72.7	0.34	12.1	24.2	0	0	33.3	0	0	32.6	12.9	188	10.1	18.1	0.32
D7	CCHF05	6.08	3.36	14.7	2.22	66.1	43.5	0.91	27.1	71.5	0.23	6.8	24.2	0	0	33.8	0	0	32.1	13.2	56	10.4	6.1	0.1
D7 (rerun)	CCHF05	6.09	3.3	14.7	2.16	65.4	43.4	0.91	27.6	71.3	0.23	7	24.1	0	0	33.9	0	0	32.2	13.2	55	11.1	4.9	0.08
D7 (rerun)	CCHF05	6.13	3.42	14.9	2.25	65.8	43.8	1.03	30.1	71.5	0.14	4.1	24.3	0	0	34	0	0	32.4	13.3	53	10.7	6.1	0.1
D9	CCHF05	5.78	5.76	13.9	1.17	20.3	41.2	3.22	55.9	71.3	1.37	23.8	24	0	0	33.7	0	0	32.6	13.3	68	10.8	3.5	0.06
D9 (rerun)	CCHF05	5.77	5.79	14	1.15	19.8	41.2	3.31	57.2	71.4	1.32	22.8	24.3	0	0	34	0.01	0.2	32.5	13.3	68	11.1	2.3	0.04
D14	CCHF05	5.67	10.3	13.7	4.17	40.5	41.2	4.45	43.2	72.7	1.65	16	24.2	0.03	0.3	33.3	0	0	34.1	14.3	260	10.1	281.8	4.97

D0	CCHF06	6.42	4.81	16	3.35	69.6	49.1	1.09	22.7	76.5	0.36	7.5	24.9	0	0	32.6	0.01	0.2	39.9	14.9	342	9.7	85.4	1.33
D1	CCHF06	5.78	10.07	14.4	8.56	85	44.8	1.06	10.5	77.5	0.42	4.2	24.9	0.01	0.1	32.1	0.02	0.2	40.7	14.9	333	9.7	63	1.09
D3	CCHF06	5.77	3.69	14.5	1.88	51	44.5	1.34	36.3	77.1	0.47	12.7	25.1	0	0	32.6	0	0	40.5	14.9	317	9.9	41.5	0.72
D5	CCHF06	6.09	2.56	15.2	0.96	37.5	46.9	1.18	46.1	77	0.42	16.4	25	0	0	32.4	0	0	40	14.8	323	9.9	16.4	0.27
D7	CCHF06	5.93	3.25	14.7	0.49	15.1	46	2.31	71.1	77.6	0.45	13.8	24.8	0	0	32	0	0	40.7	14.9	295	9.6	45.1	0.76
D9	CCHF06	6.06	7.48	15.2	1.52	20.3	47	5.02	67.1	77.6	0.94	12.6	25.1	0	0	32.3	0	0	40.8	14.9	269	10.4	66.1	1.09
D14	CCHF06	6.29	8.39	15.7	3.53	42.1	47.5	3.66	43.6	75.5	1.2	14.3	25	0	0	33.1	0	0	38.4	14.8	331	9.9	67.3	1.07
D28	CCHF06	6.39	4.8	16	2.65	55.2	48.2	1.59	33.1	75.4	0.55	11.5	25	0.01	0.2	33.2	0	0	38	15	304	10.1	77.3	1.21
D0	CCHF07	6.36	9.16	14.6	7.73	84.3	44.8	1.04	11.4	70.4	0.39	4.3	23	0	0	32.6	0	0	38.3	16.3	432	9.4	76.3	1.2
D1	CCHF07	5.75	16.42	13.2	14.67	89.3	41	1.05	6.4	71.3	0.67	4.1	23	0	0	32.2	0.03	0.2	39.1	16.4	370	9.8	59.2	1.03
D3	CCHF07	5.66	6.76	13	4.8	71	39.7	1.49	22	70.1	0.47	7	23	0	0	32.7	0	0	39.2	16.2	252	10.1	15.8	0.28
D3 (rerun)	CCHF07	5.63	6.85	13.1	4.82	70.3	39.3	1.47	21.5	69.8	0.56	8.2	23.3	0	0	33.3	0	0	39.1	16.2	258	10.4	15.8	0.28
D5	CCHF07	5.7	2.45	13.3	1.07	43.7	40.1	1	40.8	70.4	0.38	15.5	23.3	0	0	33.2	0	0	38.2	15.5	201	10.9	12.5	0.22
D7	CCHF07	5.75	6.85	13.3	1.24	18.1	40.6	3.8	55.5	70.6	1.8	26.3	23.1	0	0	32.8	0.01	0.1	38.2	15.6	79	16.1	11.5	0.2
D7 (rerun)	CCHF07	5.78	6.99	13.2	1.26	18.1	40.8	3.73	53.4	70.6	1.98	28.3	22.8	0.01	0.1	32.4	0.01	0.1	37.8	15.5	71	16.2	8.7	0.15
D7 (rerun)	CCHF07	5.72	6.93	13.2	1.23	17.8	40.3	3.76	54.3	70.5	1.93	27.8	23.1	0	0	32.8	0.01	0.1	38.4	15.5	86	15.4	7.4	0.13
D9	CCHF07	5.29	11.4	12.2	2.24	19.6	37.7	7.19	63.1	71.3	1.94	17	23.1	0	0	32.4	0.03	0.3	40	16.3	190	11.9	56.6	1.07
D14	CCHF07	5.47	7.61	12.6	4.27	56.2	39.3	2.14	28.1	71.8	1.19	15.6	23	0	0	32.1	0.01	0.1	41.7	17	588	9.7	157.5	2.88
D0	CCHF08	5.35	7.7	12.8	5.3	68.8	38.9	1.74	22.6	72.7	0.64	8.3	23.9	0.02	0.3	32.9	0	0	40.1	15.6	338	11.2	105.9	1.98
D1	CCHF08	5	10.27	12	8.22	80	37.3	1.58	15.4	74.6	0.36	3.5	24	0.06	0.6	32.2	0.05	0.5	40	15.5	338	11.3	90	1.8
D3	CCHF08	5.21	7.33	12.5	5.23	71.4	39	1.55	21.1	74.9	0.51	7	24	0.04	0.5	32.1	0	0	40.4	15.4	358	11.7	91.7	1.76
D5	CCHF08	4.83	4.61	11.7	2.74	59.4	36.1	1.32	28.6	74.7	0.52	11.3	24.2	0.03	0.7	32.4	0	0	40.8	15.6	332	11.2	76.8	1.59
D7	CCHF08	5.09	4.7	12.2	2.57	54.6	37.4	1.63	34.7	73.5	0.45	9.6	24	0.05	1.1	32.6	0	0	41	15.9	340	12	89.6	1.76
D9	CCHF08	5.21	8.29	12.6	4.4	53.1	39.2	3.08	37.2	75.2	0.73	8.8	24.2	0.07	0.8	32.1	0.01	0.1	41.4	15.7	294	12.4	69.8	1.34
D14	CCHF08	5.26	11.21	12.8	7.69	68.6	39.4	2.2	19.6	74.9	1.29	11.5	24.3	0.03	0.3	32.5	0	0	42.5	16.3	387	11	191.5	3.64
D28	CCHF08	5.53	6.03	13.4	3.58	59.4	41.6	1.69	28	75.2	0.72	11.9	24.2	0.04	0.7	32.2	0	0	40	15.3	360	11	108.9	1.97

Supplemental Table 2: Complete Blood Chemistry and Hematology for Study 2

Indian Origin

Study ID	Sex	Age (Yrs)	Group
CCHF09	Female	12.0	Sham
CCHF10	Male	2.1	
CCHF11	Female	12.2	
CCHF12	Male	2.5	
CCHF13	Female	11.3	
CCHF14	Female	10.0	
CCHF15	Female	11.3	repNP + repGPC
CCHF16	Male	2.5	
CCHF17	Female	12.0	
CCHF18	Male	2.1	
CCHF19	Female	12.2	
CCHF20	Female	11.9	

Study Day	Animal ID	BUN (mg/dL)	Creatinine (mg/dL)	ALT (U/L)	ALP (U/L)	AST (U/L)	T Bilirubin (mg/dL)	Glucose (mg/dL)	Calcium (mg/dL)	T Protein (g/dL)	Albumin (g/dL)	Globulin (g/dL)	Na+ (mmol/L)	K+ (mmol/L)	CL- (mmol/L)	tC02 (mmol/L)	Hem	Lip	Ict
D-70	CCHF09	12	1.1	50	75	48	0.3	59	10.4	7.0	5.3	1.7	145	4.6	119	26	0	0	0
D-63	CCHF09	13	1.3	63	89	53	0.3	55	10.5	6.7	5.0	1.7	139	4.5	117	23	1	0	0
D-56	CCHF09	13	1.0	45	81	44	0.3	62	10.1	6.7	4.9	1.8	147	4.2	112	29	1	0	0
D-49	CCHF09	13	0.9	33	88	44	0.3	63	10.9	6.9	5.1	1.8	142	4.6	111	29	0	0	0
D-42	CCHF09	10	0.9	29	86	45	0.3	62	10.2	7.0	5.0	2.0	141	4.7	117	25	0	0	0
D-35	CCHF09	11	0.8	40	76	42	0.3	62	10.6	7.0	5.1	1.9	145	4.7	119	27	0	0	0
D-28	CCHF09	12	1.1	53	83	47	0.3	52	10.8	7.1	5.0	2.0	150	4.7	113	27	1	0	0
D-21	CCHF09	12	1.1	31	85	47	0.3	60	10.5	6.8	4.9	1.9	148	4.3	113	29	0	0	0
D-14	CCHF09	12	1.1	33	72	45	0.3	53	10.5	7.0	5.1	1.9	150	4.3	120	27	0	0	0
D-7	CCHF09	11	1.3	52	79	57	0.3	75	11.4	6.8	5.0	1.8	145	4.7	114	29	0	0	0
D0	CCHF09	12	1.2	47	77	52	0.3	71	10.6	6.5	4.8	1.7	147	4.0	109	25	0	0	0
D1	CCHF09	13	1.1	48	85	50	0.4	59	10.6	6.9	5.0	1.9	146	4.4	111	25	0	0	0
D3	CCHF09	12	0.9	48	86	49	0.3	67	10.2	6.4	4.6	1.8	144	3.9	110	27	0	0	0
D5	CCHF09	12	1.0	38	89	68	0.2	76	9.8	6.4	4.6	1.8	146	3.5	112	24	0	0	0
D7	CCHF09	14	0.8	90	113	315	0.4	70	9.5	6.3	4.4	1.9	143	3.8	108	26	0	0	0
D-70	CCHF10	10	0.6	25	622	44	0.4	82	10.7	7.0	5.4	1.6	145	4.4	119	29	0	0	0
D-63	CCHF10	13	1.0	24	537	38	0.4	75	10.6	6.9	5.2	1.7	144	4.6	115	26	0	0	0
D-56	CCHF10	12	0.6	22	548	40	0.4	83	10.9	7.0	5.0	2.0	147	4.2	108	28	0	0	0
D-49	CCHF10	9	0.5	28	612	36	0.4	88	11.0	7.0	5.1	1.9	145	4.7	111	31	0	0	0
D-42	CCHF10	14	0.8	23	580	42	0.3	86	10.7	7.0	5.0	1.9	147	4.6	114	30	0	0	0
D-35	CCHF10	12	0.3	30	581	44	0.3	87	10.7	6.9	5.0	1.9	145	4.6	114	30	0	0	0
D-28	CCHF10	14	0.8	24	553	43	0.4	86	10.8	7.3	5.2	2.1	149	4.6	110	30	0	0	0
D-21	CCHF10	10	0.7	25	574	37	0.2	82	10.8	7.1	5.0	2.1	149	4.3	113	28	0	0	0
D-14	CCHF10	19	0.8	23	492	35	0.3	98	10.8	7.1	5.1	2.0	151	3.9	112	27	0	0	0
D-7	CCHF10	12	0.7	36	545	49	0.1	87	11.0	6.6	5.0	1.6	145	3.9	109	29	0	0	0
D0	CCHF10	11	0.9	28	522	37	0.1	89	10.2	6.9	4.9	2.0	150	3.9	113	26	0	0	0
D1	CCHF10	16	0.6	32	452	41	0.2	78	10.3	6.6	4.8	1.8	151	4.0	115	28	0	0	0
D3	CCHF10	15	0.8	45	462	52	0.1	83	9.5	6.6	4.7	1.9	147	3.8	108	27	0	0	0
D5	CCHF10	14	1.0	64	503	97	0.2	85	8.9	6.5	4.5	2.0	146	3.5	107	25	0	0	0
D7	CCHF10	15	1.1	89	515	211	0.1	82	9.4	6.0	4.0	2.0	143	3.4	107	30	0	0	0
D-70	CCHF11	11	0.7	62	92	38	0.3	64	10.0	7.1	4.6	2.5	141	3.5	117	28	0	0	0
D-63	CCHF11	12	1.0	50	106	40	0.3	71	10.2	7.4	4.5	2.9	140	3.9	113	24	1	0	0
D-56	CCHF11	10	0.7	52	107	34	0.3	76	10.1	7.0	4.3	2.7	144	3.7	111	28	1	0	0
D-49	CCHF11	11	1.0	37	107	35	0.3	74	10.5	7.5	4.6	2.9	141	3.8	111	27	0	0	0
D-42	CCHF11	11	0.9	22	89	28	0.3	73	10.3	7.6	4.5	3.1	143	3.9	115	27	0	0	0
D-35	CCHF11	11	0.9	51	85	42	0.3	63	10.2	7.2	4.4	2.8	140	4.8	113	26	2	0	0
D-28	CCHF11	13	1.1	36	87	31	0.3	73	10.3	7.6	4.4	3.2	148	3.9	112	29	0	0	0
D-21	CCHF11	10	1.2	35	84	35	0.3	68	10.0	7.1	4.4	2.7	145	3.7	114	27	0	0	0
D-14	CCHF11	13	1.0	41	85	33	0.4	79	10.3	7.5	4.6	2.9	148	3.6	114	26	0	0	0
D-7	CCHF11	11	1.0	229	106	103	0.4	86	10.5	7.2	4.6	2.5	141	3.8	109	28	0	1	0
D0	CCHF11	11	0.9	71	112	39	0.2	73	10.1	7.1	4.3	2.8	143	3.8	108	24	1	0	0
D1	CCHF11	14	1.0	61	114	37	0.2	80	10.4	7.2	4.4	2.8	145	4.0	112	27	0	0	0
D3	CCHF11	10	0.8	61	109	57	0.3	79	9.7	7.1	4.2	2.9	145	4.0	113	27	0	0	0
D5	CCHF11	11	1.3	83	114	61	0.2	86	9.9	6.8	4.1	2.6	144	4.0	109	27	0	0	0
D7	CCHF11	8	0.6	133	113	119	0.3	61	9.5	6.3	4.0	2.3	143	3.8	112	32	0	0	0
D-70	CCHF12	14	0.7	36	657	42	0.3	65	10.8	6.1	4.9	1.2	143	4.5	116	28	0	0	0
D-63	CCHF12	16	0.5	32	734	36	0.3	62	10.8	6.4	5.0	1.4	145	3.9	116	23	0	0	0
D-56	CCHF12	17	0.5	36	661	38	0.4	67	10.4	6.3	4.8	1.5	146	3.7	107	27	0	0	0
D-49	CCHF12	12	0.6	31	587	38	0.3	76	10.8	6.4	5.0	1.4	146	4.2	111	28	0	0	0
D-42	CCHF12	18	0.5	25	530	36	0.3	71	10.8	6.6	5.0	1.6	146	4.5	112	30	0	0	0
D-35	CCHF12	15	0.5	30	582	37	0.3	70	10.6	6.5	5.0	1.5	146	4.4	114	27	0	0	0
D-28	CCHF12	15	0.8	25	631	37	0.3	69	11.0	6.6	5.1	1.6	150	4.0	107	29	0	0	0
D-21	CCHF12	13	0.6	30	619	36	0.3	71	10.3	6.3	4.8	1.5	151	4.0	116	24	0	0	0
D-14	CCHF12	18	0.6	28	577	35	0.3	65	10.5	6.8	5.2	1.6	151	3.6	115	26	0	0	0
D-7	CCHF12	11	1.0	34	585	46	0.4	79	11.0	6.4	5.0	1.4	150	3.8	114	28	0	0	0
D0	CCHF12	13	0.5	36	534	35	0.3	78	10.5	6.4	4.9	1.5	150	3.6	112	25	0	0	0
D1	CCHF12	17	0.6	35	520	37	0.4	73	10.5	6.4	5.0	1.5	146	4.0	113	24	0	0	0
D3	CCHF12	18	0.3	36	541	45	0.3	70	9.6	6.0	4.5	1.5	147	4.0	111	25	0	0	0
D5	CCHF12	14	0.9	59	479	55	0.4	79	10.0	6.0	4.6	1.5	147	4.0	108	26	0	0	0
D7	CCHF12	16	0.6	72	494	71	0.3	61	10.3	5.6	4.2	1.4	145	4.2	~~~	32	0	0	0
D7 (rerun)	CCHF12	16	0.6	72	497	68	0.3	59	10.6	5.5	4.2	1.3	150	3.7	112	29	0	0	0
D-70	CCHF13	15	1.2	245	89	69	0.4	56	10.4	6.8	5.0	1.8	147	4.2	115	27	0	0	0
D-63	CCHF13	15	1.0	336	104	129	0.4	61	10.5	6.6	4.8	1.9	144	4.3	115	26	0	0	0
D-56	CCHF13	15	1.0	165	101	50	0.4	62	10.6	6.6	4.8	1.8	146	4.1	115	29	0	0	0
D-49	CCHF13	16	1.1	82	83	49	0.5	54	10.0	6.8	4.8	2.0	147	4.3	108	27	0	0	0
D-42	CCHF13	15	1.1	84	87	45	0.3	60	10.6	6.6	4.7	1.9	148	4.2	109	29	0	0	0
D-35	CCHF13	15	1.1	119	84	53	0.3	58	10.6	7.0	4.6	2.4	147	4.3	114	28	1	0	0
D-28	CCHF13	16	1.2	119	89	47	0.4	53	10.5	6.7	4.6	2.2	149	4.5	111	29	1	0	0
D-21	CCHF13	15	1.1	100	93	50	0.3	60	10.4	6.8	4.6	2.1	147	4.2	113	28	0	0	0
D-14	CCHF13	17	0.9	81	73	43	0.2	51	9.9	6.5	4.5	2.0	143	4.1	107	25	1	0	0
D-7	CCHF13	14	1.2	113	92	63	0.3	58	11.0	6.3	4.4	1.9	145	3.9	107	31	0	0	0
D0	CCHF13	14	1.1	88	90	42	0.3	50	10.2	6.3	4.4	1.9	149	4.6	113	28	0	0	0
D1	CCHF13	17	1.2	89	85	58	0.6	54	10.3	6.2	4.4	1.9	145	4.1	107	28	0	0	0
D3	CCHF13	13	0.9	120	86	105	0.5	84	9.1	5.9	4.1	1.8	143	4.2	109	24	0	0	0
D5	CCHF13	12	1.1	147	83	131	0.4	81	9.7	5.9	4.1	1.8	143	4.1	107	27	0	0	0
D7	CCHF13	10	0.9	154	112	157	0.4	61	9.7	5.8	3.9	1.9	144	4.3	111	28	0	0	0
D-70	CCHF14	16	0.8	41	123	38	0.4	50	10.2	6.9	5.3	1.6	146	3.9	115	26	0	0	0
D-63	CCHF14	16	1.0	51	138	38	0.3	71	10.6	7.0	5.1	1.9	146	4.2	120	24	0	0	0
D-56	CCHF14	12	1.1	52	126	39	0.4	77	10.3	6.8	5.0	1.8	147	3.8	111	28	0	0	0
D-49	CCHF14	16	1.2	41	132	36	0.1	73	10.9	7.0	4.9	2.1	147	3.8	108	28	0	0	0
D-42	CCHF14	15	1.1	34	125	32	0.3	74	10.7	6.9	5.2	1.7	145	3.8	115	27	0	0	0
D-35	CCHF14	14	1.1	43	116	31	0.1	88	10.4	7.0	4.9	2.1	147	4.0	116	24	0	0	0
D-28	CCHF14	14	1.1	35	118	34	0.2	75	10.7	6.5	4.8	1.7	147	4.0	110	29	0	0	0
D-21	CCHF14	12	1.2	40	128	34	0.3	80	10.5	6.7	4.9	1.8	149	4.2	113	26	0	0	0
D-14	CCHF14	13	1.0	40	110	35	0.4	88	10.0	6.6	5.0	1.6	146	3.6	111	25	0	0	0
D-7	CCHF14	13	0.7	57	138	38	0.3	70	10.7	6.8	5.1	1.8	145	4.0	111	29	0	0	0
D0	CCHF14	11	1.2	50	124	36	0.3	75	10.5	7.0	5.0	2.1	151	4.8	117	23	0	0	0
D1	CCHF14	16	1.3	49	139	39	0.3	94	10.4	6.8	4.9	1.9	147	4.0	111	25	0	0	0
D3	CCHF14	14	1.5	61	171	68	0.4	113	9.6	6.7	4.6	2.1	143	3.9	113	21	0	0	0
D5	CCHF14	9	1.5	81	200	81	0.4	104	9.7	6.2	4.4	1.8	142	3.9	110	27	0	0	0
D7	CCHF14	10	0.8	109	229	87	<0.1	77	9.3	6.0	4.1	1.8	149	4.2	111	29	0	0	0
D-70	CCHF15	14	1.2	76	82	42	0.3	48	11.1	7.0	5.5	1.5	147	4.1	121	26	0	0	0
D-63	CCHF15	13	1.3	53	90	40	0.4	63	11.0	7.1	5.4	1.7	143	4.2	118	24	0	0	0
D-56	CCHF15	13	1.0	64	96	44	0.4	65	11.4	6.9	5.3	1.6	147	4.1	111	29	0	0	0
D-49	CCHF15	17	1.5	43	93	32	0.4	55	11.0	7.2	5.3	1.9	148	4.1	116	25	1	0	0
D-42	CCHF15	16	1.3	79	76	48	0.3	63	11.1	7.0	5.1	1.8	149	4.2	118	27	0	0	0
D-35	CCHF15	15	1.1	62	81	38	0.3	70	11.1	7.0	5.2	1.7	146	4.0	116	26	0	0	0
D-28	CCHF15	14	1.1	51	85	45	0.4	59	10.6	7.0	5.2	1.8	147	4.3	117	28	1	0	0
D-21	CCHF15	14	1.0	56	99	44	0.3	69	10.8	7.0	5.0	2.0	152	4.4	115	25	0	0	0
D-14	CCHF15	18	1.1	102	85	48	0.3	63	11.2	7.2	5.3	1.9	151	4.3	120	25	0	0	0
D-7	CCHF15	13	1.4	61	83	44	0.3	78	11.4	7.2	5.2	2.0	148	4.3	110	27	0	0	0
D0	CCHF15	13	1.4	48	78	41	0.5	60	10.8	6.9	5.1	1.9	152	4.5	116	27	0	0	0
D1	CCHF15	15	1.1	62	84	64	0.4	72	10.7	6.9	5.1	1.7	148	4.2	111	29	0	0	0
D3	CCHF15	12	1.3	106	93	59	0.3	72	10.7	7.3	5.0	2.2	145	3.9	112	24	0	0	0
D5	CCHF15	14	1.0	82	103	61	0.4	71	10.6	7.0	5.0	2.0	146	3.9	111	26	0	0	0

D7	CCHF15	12	0.9	68	96	55	0.4	79	10.3	7.0	4.9	2.0	147	3.9	112	25	0	0	0
D-70	CCHF16	9	0.5	30	552	36	0.4	74	11.1	6.5	5.2	1.3	149	4.0	116	26	0	0	0
D-63	CCHF16	11	0.6	24	510	34	0.4	76	10.8	7.0	5.1	1.9	147	4.6	114	23	0	0	0
D-56	CCHF16	10	0.7	22	564	35	0.3	73	11.0	6.7	5.1	1.6	147	4.3	115	24	0	0	0
D-49	CCHF16	12	0.9	27	522	35	0.4	67	11.3	7.1	5.3	1.8	149	4.1	109	24	0	0	0
D-42	CCHF16	14	0.5	26	477	40	0.4	67	11.2	6.8	5.1	1.7	151	4.5	115	26	0	0	0
D-35	CCHF16	11	0.8	30	495	35	0.4	91	11.2	6.9	5.1	1.8	149	4.3	115	25	0	0	0
D-28	CCHF16	11	1.2	53	474	39	0.4	76	10.9	6.8	5.0	1.7	152	4.4	115	27	0	0	0
D-21	CCHF16	14	0.8	28	422	37	0.3	65	10.7	6.8	4.9	2.0	151	4.5	112	24	0	0	0
D-14	CCHF16	15	0.6	31	456	37	0.4	62	11.0	7.0	5.2	1.9	151	4.2	113	24	0	0	0
D-7	CCHF16	9	0.6	47	512	45	0.4	83	11.2	6.7	5.0	1.7	148	4.4	109	27	0	0	0
D0	CCHF16	11	0.7	32	469	37	0.3	61	10.5	6.5	4.9	1.6	153	4.1	119	27	0	0	0
D1	CCHF16	10	0.7	39	415	41	0.4	75	10.7	6.5	4.9	1.5	149	4.2	109	26	0	0	0
D3	CCHF16	13	0.7	39	361	33	0.3	65	10.4	6.6	4.8	1.8	147	3.9	109	26	0	0	0
D5	CCHF16	11	1.0	41	368	30	0.4	64	10.7	6.7	5.0	1.7	147	4.0	107	28	0	0	0
D7	CCHF16	9	0.7	50	397	40	0.3	68	10.8	6.7	4.8	1.8	147	4.1	110	27	0	0	0
D-70	CCHF17	13	1.0	70	73	45	0.3	68	9.6	7.0	5.0	2.0	151	3.5	113	28	0	0	0
D-63	CCHF17	11	1.1	41	91	38	0.3	82	10.7	7.3	5.3	2.0	147	4.1	117	26	0	0	0
D-56	CCHF17	11	1.0	47	90	39	0.3	74	10.2	7.1	5.3	1.8	145	3.8	112	28	0	0	0
D-49	CCHF17	12	0.9	57	89	41	0.3	81	10.2	7.1	5.0	2.0	145	3.9	112	25	0	0	0
D-42	CCHF17	16	0.6	58	73	44	0.3	70	10.6	7.2	5.0	2.2	144	3.7	114	26	0	0	0
D-35	CCHF17	16	0.9	43	71	38	0.3	70	11.0	7.1	4.9	2.2	150	4.1	113	27	2	0	0
D-28	CCHF17	15	1.3	58	77	41	0.3	70	10.0	7.1	5.0	2.1	146	3.9	109	26	0	0	0
D-21	CCHF17	10	0.9	67	90	48	0.3	88	10.6	7.2	4.9	2.3	147	3.8	113	27	0	0	0
D-14	CCHF17	16	0.9	59	75	41	0.3	75	10.5	7.2	5.0	2.2	148	3.9	114	27	1	0	0
D-7	CCHF17	12	1.1	60	78	50	0.3	83	10.3	7.0	4.9	2.1	147	4.0	109	28	0	0	0
D0	CCHF17	11	1.0	52	78	38	0.3	72	10.3	7.2	5.0	2.2	149	4.1	115	28	0	0	0
D1	CCHF17	15	1.0	57	81	49	0.4	75	10.3	6.9	4.9	2.0	145	3.7	110	27	0	0	0
D3	CCHF17	13	0.8	73	103	110	0.4	76	9.3	6.5	4.5	2.0	146	3.5	112	24	0	0	0
D5	CCHF17	14	1.1	214	181	295	0.4	72	8.9	6.4	4.3	2.1	143	3.9	111	24	0	0	0
D7	CCHF17	7	1.0	184	271	162	0.2	70	9.8	6.4	4.1	2.2	148	4.2	112	31	0	0	0
D-70	CCHF18	11	0.9	32	792	43	0.4	74	10.9	6.8	5.0	1.8	154	4.3	118	22	0	0	0
D-63	CCHF18	12	0.9	27	712	35	0.4	84	11.1	6.9	5.1	1.8	149	4.1	117	23	0	0	0
D-56	CCHF18	4	0.2	11	253	16	0.3	30	4.1	2.3	1.4	0.9	<100	<1.5	<75	7	0	0	0
D-56 (rerun)	CCHF18	13	0.5	29	739	36	0.4	76	11..1	6.8	4.8	2.0	151	4.2	112	24	0	0	0
D-49	CCHF18	13	0.7	28	822	37	0.4	70	11.3	6.9	5.0	1.9	148	4.2	111	24	0	0	0
D-42	CCHF18	16	0.6	25	737	39	0.3	71	11.1	6.9	4.8	2.0	153	4.3	117	25	0	0	0
D-35	CCHF18	15	0.7	31	793	40	0.3	78	10.8	6.9	4.9	2.0	150	4.2	117	22	0	0	0
D-28	CCHF18	12	0.6	28	742	38	0.3	65	11.0	6.7	4.8	1.9	151	4.5	114	27	0	0	0
D-21	CCHF18	14	0.7	27	673	35	0.2	67	10.9	6.6	4.6	2.0	153	4.4	113	25	0	0	0
D-14	CCHF18	14	1.0	26	722	34	0.2	76	11.3	7.1	5.0	2.1	153	4.3	116	24	0	0	0
D-7	CCHF18	12	1.1	30	780	41	0.2	77	11.2	6.6	4.9	1.7	148	4.1	111	26	0	0	0
D0	CCHF18	11	0.7	39	804	42	0.3	64	11.1	6.8	5.0	1.9	152	4.4	118	26	0	0	0
D1	CCHF18	13	0.9	37	724	39	0.3	71	10.9	6.6	4.8	1.8	154	4.4	118	26	0	0	0
D3	CCHF18	18	0.5	44	676	43	0.1	68	10.0	6.6	4.7	1.9	148	3.7	114	25	0	0	0
D5	CCHF18	13	1.0	48	681	44	0.6	73	10.4	6.6	4.8	1.8	146	4.0	111	26	0	0	0
D7	CCHF18	12	0.6	45	734	45	0.3	61	11.0	6.7	4.8	1.9	150	4.1	114	27	0	0	0
D-70	CCHF19	17	1.3	65	73	37	0.3	64	10.4	7.0	5.2	1.8	151	4.2	117	26	0	0	0
D-63	CCHF19	18	1.3	75	87	42	0.3	68	10.2	6.8	5.3	1.5	145	4.0	118	25	0	0	0
D-56	CCHF19	17	1.1	44	87	29	0.3	64	10.2	6.8	5.3	1.5	148	4.1	119	27	0	0	0
D-49	CCHF19	17	1.2	41	90	32	0.3	64	10.6	6.8	5.2	1.7	149	3.9	113	26	0	0	0
D-42	CCHF19	17	1.1	81	83	45	0.3	63	10.5	6.9	5.1	1.8	151	4.1	119	26	0	0	0
D-35	CCHF19	18	1.1	52	82	37	0.3	69	10.4	6.7	5.0	1.7	149	4.2	115	26	0	0	0
D-28	CCHF19	18	1.3	53	73	34	0.3	67	10.2	6.9	5.1	1.8	151	4.1	114	27	0	0	0
D-21	CCHF19	14	1.4	58	85	37	0.3	72	10.2	6.6	5.0	1.6	147	4.1	121	26	0	0	0
D-14	CCHF19	21	1.1	66	79	35	0.3	64	10.6	7.1	5.4	1.7	152	4.3	116	28	0	0	0
D-7	CCHF19	16	1.5	59	80	32	0.4	64	10.3	6.7	5.2	1.5	145	4.0	113	29	0	0	0
D0	CCHF19	18	1.5	61	77	38	0.3	54	10.3	7.1	5.2	1.9	151	4.2	119	27	0	0	0
D1	CCHF19	18	1.4	67	73	50	0.4	71	10.1	6.6	5.0	1.6	150	4.0	118	26	0	0	0
D3	CCHF19	11	1.3	69	94	56	0.4	82	8.9	6.3	4.8	1.5	147	3.7	113	26	0	0	0
D5	CCHF19	11	1.4	66	88	69	0.4	73	9.4	6.1	4.7	1.5	143	3.5	108	28	0	0	0
D7	CCHF19	10	1.1	54	85	40	0.3	70	9.3	6.5	4.8	1.7	149	3.9	114	28	0	0	0
D-70	CCHF20	15	0.9	38	120	34	0.4	65	9.5	6.4	4.7	1.7	148	3.6	113	24	0	0	0
D-63	CCHF20	16	1.0	28	145	33	0.4	58	9.6	6.4	4.9	1.5	143	3.3	116	23	0	0	0
D-56	CCHF20	16	0.9	44	126	33	0.4	61	9.7	6.5	5.0	1.5	146	3.6	115	25	0	0	0
D-49	CCHF20	15	1.1	42	131	37	0.5	61	9.8	6.6	5.0	1.7	146	3.5	113	24	0	0	0
D-42	CCHF20	15	0.9	32	129	32	0.3	69	10.2	6.7	4.9	1.8	153	3.9	115	27	0	0	0
D-35	CCHF20	14	0.7	51	133	35	0.3	66	9.9	6.8	4.9	1.8	150	4.0	115	27	0	0	0
D-28	CCHF20	15	1.1	42	123	29	0.4	65	9.8	6.7	5.0	1.7	150	4.0	113	27	0	0	0
D-21	CCHF20	15	0.7	46	125	43	0.3	75	10.0	6.8	4.9	1.9	151	3.9	117	26	0	0	0
D-14	CCHF20	19	0.8	40	114	39	0.4	66	10.2	6.9	5.1	1.8	150	4.1	115	23	1	0	0
D-7	CCHF20	13	0.7	42	112	36	0.4	67	9.7	6.6	4.9	1.6	149	4.0	113	28	0	0	0
D0	CCHF20	14	1.0	39	115	35	0.3	66	9.6	6.8	5.0	1.8	150	3.9	119	27	0	0	0
D1	CCHF20	14	1.0	36	109	37	0.4	65	9.4	6.4	4.8	1.7	149	3.6	118	26	0	0	0
D3	CCHF20	15	1.0	43	129	51	0.4	65	8.6	6.8	5.0	1.8	144	4.0	113	24	0	0	0
D5	CCHF20	11	1.1	36	122	47	0.5	87	9.4	6.5	4.7	1.8	142	3.1	113	23	0	0	0
D7	CCHF20	13	1.0	53	197	127	0.5	69	9.1	6.3	4.5	1.8	143	3.2	110	26	0	0	0

Study Day	Animal ID	RBC (M/uL)	WBC (K/uL)	HGB (g/dL)	NEUT# (K/uL)	NEUT% (%)	HCT (%)	LYMPH# (K/uL)	LYMPH% (%)	MCV (fL)	MONO# (K/uL)	MONO% (%)	MCH (pg)	EOS# (K/uL)	EOS% (%)	MCHC (g/dL)	BASO# (K/uL)	BASO% (%)	RDW SD (fL)	RDW CV (%)	PLT (K/uL)	MPV (fL)	RET# (K/uL)	RET% (%)
D-70	CCHF09	5.98	6.67	14	4.29	64.3	42	1.78	26.7	70.2	0.6	9	23.4	0	0	33.3	0	0	37.7	16	411	10.8	76.5	1.28
D-63	CCHF09	5.76	8.78	13.3	5.38	61.3	40.4	2.78	31.7	70.1	0.59	6.7	23.1	0.03	0.3	32.9	0	0	37.3	15.5	467	11.7	103.1	1.79
D-56	CCHF09	5.73	4.91	13.4	2.5	50.9	40.9	1.94	39.5	71.4	0.45	9.2	23.4	0.01	0.2	32.8	0.01	0.2	38.8	15.9	427	11.6	88.8	1.55
D-49	CCHF09	5.4	5.62	12.7	3.14	55.8	38.7	1.92	34.2	71.7	0.55	9.8	23.5	0.01	0.2	32.8	0	0	38.7	15.6	507	11.2	94	1.74
D-42	CCHF09	5.91	5.06	13.7	2.53	50	42.3	2.14	42.3	71.6	0.39	7.7	23.2	0	0	32.4	0	0	37.8	15.8	467	11.1	87.5	1.48
D-35	CCHF09	5.6	4.95	12.9	2.43	49.1	40.1	2.08	42	71.6	0.43	8.7	23	0.01	0.2	32.2	0	0	38.3	15.4	314	11.9	85.1	1.52
D-28	CCHF09	5.95	4.5	13.9	2.27	50.4	42.7	1.78	39.6	71.8	0.45	10	23.4	0	0	32.6	0	0	37.7	15.6	484	11.2	97	1.63
D-21	CCHF09	5.57	4.16	12.8	1.78	42.8	40.2	1.94	46.6	72.2	0.44	10.6	23	0	0	31.8	0	0	37.4	15	487	11.5	91.3	1.64
D-14	CCHF09	5.73	6.31	13.7	4.45	70.6	40.8	1.32	20.9	71.2	0.5	7.9	23.9	0.04	0.6	33.6	0	0	36.7	14.9	405	11	85.4	1.49
D-7	CCHF09	5.95	5.05	13.9	2.7	53.5	43.3	1.89	37.4	72.8	0.44	8.7	23.4	0.02	0.4	32.1	0	0	37.3	14.9	465	10.9	105.3	1.77
D-7 (rerun)	CCHF09	5.94	5.17	14.1	2.7	52.2	43.4	2.03	39.3	73.1	0.43	8.3	23.7	0.01	0.2	32.5	0	0	37.7	14.9	470	11	114	1.92
D0	CCHF09	5.81	4.66	14	2.61	56.1	41.6	1.54	33	71.6	0.49	10.5	24.1	0.02	0.4	33.7	0	0	36.4	14.7	397	10.2	101.1	1.74
D1	CCHF09	5.95	8.93	14	7.18	80.4	42.9	0.98	11	72.1	0.77	8.6	23.5	0	0	32.6	0	0	36.4	14.7	393	10.4	95.8	1.61
D3	CCHF09	5.55	4.09	13	1.95	47.7	39.6	1.55	37.9	71.4	0.54	13.2	23.4	0.05	1.2	32.8	0	0	36.8	14.8	379	11.1	57.2	1.03
D5	CCHF09	5.09	2.42	12	1.56	64.5	36.2	0.68	28.1	71.1	0.16	6.6	23.6	0.02	0.8	33.1	0	0	36.5	14.9	289	11.3	35.1	0.69
D7	CCHF09	5.42	2.17	12.7	1.24	57.1	37.6	0.82	37.8	69.4	0.11	5.1	23.4	0	0	33.8	0	0	34.9	14.7	200	11.4	15.2	0.28
D7 (rerun)	CCHF09	5.43	2.19	12.9	1.26	57.5	37.3	0.77	35.2	68.7	0.16	7.3	23.8	0	0	34.6	0	0	34.4	14.6	195	11.4	18.5	0.34
D-70	CCHF10	6.05	13.37	13.6	5.76	43.1	40	6.87	51.4	66.1	0.71	5.3	22.5	0.01	0.1	34	0.02	0.1	33.9	15.5	281	10.4	26.6	0.44
D-63	CCHF10	5.79	14.23	12.3	8.05	56.5	38	5.56	39.1	65.6	0.61	4.3	21.2	0	0	32.4	0.01	0.1	34.3	15.3	303	11.5	57.3	0.99
D-56	CCHF10	5.94	9.13	12.9	3.82	41.9	39.6	4.75	52	66.7	0.56	6.1	21.7	0	0	32.6	0	0	35.6	15.7	304	11.2	45.1	0.76
D-49	CCHF10	5.68	13.88	12.7	8.01	57.7	38.4	5.18	37.3	67.6	0.68	4.9	22.4	0	0	33.1	0.01	0.1	36.4	15.6	348	10.9	59.6	1.05
D-42	CCHF10	5.98	9.14	13.1	2.82	30.9	40.3	5.78	63.2	67.4	0.51	5.6	21.9	0.02	0.2	32.5	0.01	0.1	35.8	15.4	305	10.7	41.9	0.7
D-35	CCHF10	5.58	10.87	12	3.53	32.5	37.7	6.74	62	67.6	0.59	5.4	21.5	0	0	31.8	0.01	0.1	36	15.4	336	10.5	38.5	0.69
D-28	CCHF10	5.98	8.66	13.1	3.36	38.9	40.4	4.68	54	67.6	0.6	6.9	21.9	0.01	0.1	32.4	0.01	0.1	35.2	15.4	349	11	44.9	0.75
D-21	CCHF10	5.8	10.58	12.5	4.34	41	39.1	5.66	53.5	67.4	0.57	5.4	21.6	0.01	0.1	32	0	0	34.8	15	425	11.4	66.7	1.15
D-14	CCHF10	5.87	11.91	13.3	5.54	46.5	39.4	5.7	47.9	67.1	0.66	5.5	22.7	0	0	33.8	0.01	0.1	34.6	15	324	10.6	27	0.46
D-7	CCHF10	5.59	12.62	12.4	5.71	45.2	38.7	5.91	46.8	69.2	0.97	7.7	22.2	0.02	0.2	32	0.01	0.1	35.4	14.9	343	10.2	51.4	0.92
D0	CCHF10	5.82	9.6	13	4.25	44.2	39.2	4.51	47	67.4	0.84	8.8	22.3	0	0	33.2	0	0	34.4	14.9	292	10.1	42.5	0.73
D1	CCHF10	5.31	16.94	11.9	12.51	73.8	35.7	3.45	20.4	67.2	0.97	5.7	22.4	0.01	0.1	33.3	0	0	34.4	14.9	285	9.9	34.5	0.65
D3	CCHF10	5.63	7.69	12.5	3.94	51.3	38	3.11	40.4	67.5	0.64	8.3	22.2	0	0	32.9	0	0	34.6	14.9	271	10.4	31.5	0.56
D5	CCHF10	5.4	4.53	11.9	2.02	44.6	35.9	2.04	45	66.5	0.47	10.4	22	0	0	33.1	0	0	34	14.8	195	10.8	15.7	0.29
D5 (rerun)	CCHF10	5.39	4.25	11.9	1.86	43.7	35.9	1.97	46.4	66.6	0.42	9.9	22.1	0	0	33.1	0	0	34.2	14.9	202	10.8	12.9	0.24
D7	CCHF10	5.15	7.3	11.4	1	13.7	33.7	4.93	67.5	65.4	1.37	18.8	22.1	0	0	33.8	0	0	32.3	14.4	63	15.5	6.7	0.13
D7 (rerun)	CCHF10	5.22	7.32	11.5	1.03	14.1	33.7	4.84	66.1	64.6	1.45	19.8	22	0	0	34.1	0	0	32.1	14.4	60	15.6	7.3	0.14
D-70	CCHF11	6.2	6.71	13.8	3.14	46.9	41.9	2.94	43.8	67.6	0.6	8.9	22.3	0.03	0.4	32.9	0	0	34.5	15.5	262	10.1	62	1
D-63	CCHF11	6.28	11.67	13.9	7.19	61.7	42.6	3.76	32.2	67.8	0.68	5.8	22.1	0.04	0.3	32.6	0	0	34.2	15.3	278	10.6	65.3	1.04
D-56	CCHF11	6.23	7.64	14	3.42	44.8	42	3.55	46.5	67.4	0.56	7.3	22.5	0.03	0.4	33.3	0.08	1	34.8	16.5	360	12.3	69.2	1.11
D-49	CCHF11	6.3	8.97	14.3	3.46	38.5	43.3	4.49	50.1	68.7	0.94	10.5	22.7	0.07	0.8	33	0.01	0.1	35	16.1	364	10	94.5	1.5
D-42	CCHF11	6.38	7.51	14.2	3.41	45.4	43.2	3.49	46.5	67.7	0.61	8.1	22.3	0	0	32.9	0	0	34.5	16.1	291	10.3	66.4	1.04
D-35	CCHF11	6.12	8.17	13.6	3.51	43	41.9	3.91	47.9	68.5	0.64	7.8	22.2	0.11	1.3	32.5	0	0	34.7	15.4	113	11.1	71.6	1.17
D-35 (rerun)	CCHF11	6.1	8.31	13.7	3.55	42.8	41.7	4.08	49.1	68.4	0.56	6.7	22.5	0.11	1.3	32.9	0.01	0.1	34.8	15.3	137	11.8	66.5	1.09
D-28	CCHF11	6.34	7.82	14.1	3.39	43.4	43.2	3.88	49.6	68.1	0.49	6.3	22.2	0.05	0.6	32.6	0.01	0.1	35.3	16.4	305	10.4	89.4	1.41
D-21	CCHF11	5.92	7.25	13	2.35	32.5	41.1	4.15	57.2	69.4	0.72	9.9	22	0.03	0.4	31.6	0	0	34.7	14.9	232	10.8	128.5	2.17
D-21 (rerun)	CCHF11	5.91	7.34	13.2	2.45	33.4	41	4.42	60.2	69.4	0.44	6	22.3	0.03	0.4	32.2	0	0	34.7	15	239	11.2	133	2.25
D-14	CCHF11	6.5	7.44	14.9	2.98	40.1	44.3	3.73	50.1	68.2	0.65	8.7	22.9	0.08	1.1	33.6	0	0	35.1	15.8	279	10.2	59.2	0.91
D-7	CCHF11	6.44	7.98	14.6	3.57	44.7	44.7	3.62	45.4	69.4	0.75	9.4	22.7	0.04	0.5	32.7	0	0	35.8	15.8	338	10	112.7	1.75

D0	CCHF11	6.24	7.73	14.3	3.35	43.3	43	3.97	51.4	68.9	0.41	5.3	22.9	0	0	33.3	0	0	34.9	15.3	75	11.4	98.6	1.58
D0 (rerun)	CCHF11	6.45	8.12	14.8	3.44	42.3	43.5	4.04	49.8	67.4	0.61	7.5	22.9	0	0	34	0.03	0.4	34.9	15.9	184	12.3	107.1	1.66
D0 (rerun)	CCHF11	6.21	7.86	14.2	3.33	42.4	42.5	3.81	48.5	68.4	0.71	9	22.9	0	0	33.4	0.01	0.1	34.9	15.3	134	11	98.1	1.58
D0 (rerun)	CCHF11	6.49	8.39	15.1	3.47	41.3	43.6	4.01	47.8	67.2	0.77	9.2	23.3	0.04	0.5	34.6	0.1	1.2	35	15.9	234	11.8	92.2	1.42
D1	CCHF11	6.04	8.59	13.7	5.98	69.6	42.1	2.02	23.5	69.7	0.58	6.8	22.7	0	0	32.5	0.01	0.1	35.6	15.1	219	9.6	84.6	1.4
D3	CCHF11	6.03	5.9	13.7	1.89	32	42.2	3.34	56.6	70	0.62	10.5	22.7	0.04	0.7	32.5	0.01	0.2	36.5	15.4	232	10.4	53.7	0.89
D5	CCHF11	6.01	3.95	13.6	1.45	36.7	41.9	2.11	53.4	69.7	0.37	9.4	22.6	0.02	0.5	32.5	0	0	35	14.8	243	9.9	34.3	0.57
D7	CCHF11	5.68	4.88	12.8	1.2	24.6	39.2	3.28	67.2	69	0.37	7.6	22.5	0.03	0.6	32.7	0	0	34.7	14.8	196	10.4	60.8	1.07
D7 (rerun)	CCHF11	5.67	4.94	12.8	1.1	22.3	38.7	2.83	57.3	68.3	1	20.2	22.6	0.01	0.2	33.1	0	0	34.4	14.8	204	10.2	61.2	1.08
D-70	CCHF12	5.65	7.01	13.1	2.02	28.8	37.6	4.49	64.1	66.5	0.5	7.1	23.2	0	0	34.8	0	0	30.8	13.8	450	10.2	29.9	0.53
D-63	CCHF12	5.41	8.48	12.9	3.35	39.5	36.3	4.5	53.1	67.1	0.62	7.3	23.8	0	0	35.5	0.01	0.1	30.6	13.6	529	10.3	70.9	1.31
D-56	CCHF12	5.84	6.09	13.6	2.3	37.8	39.8	3.48	57.1	68.2	0.3	4.9	23.3	0.01	0.2	34.2	0	0	32.5	14.1	354	10.5	66.6	1.14
D-49	CCHF12	5.5	7.03	12.9	2.74	39	38.2	3.98	56.6	69.5	0.31	4.4	23.5	0	0	33.8	0	0	33.3	14	485	10.5	60	1.09
D-42	CCHF12	5.69	7.34	13.3	2.37	32.3	38.8	4.37	59.5	68.2	0.6	8.2	23.4	0	0	34.3	0	0	33.2	14.3	493	10.4	47.8	0.84
D-35	CCHF12	5.49	6.38	12.8	1.74	27.2	38.1	4.01	62.9	69.4	0.63	9.9	23.3	0	0	33.6	0	0	33.5	14.2	469	10.1	54.9	1
D-28	CCHF12	5.78	5.43	13.7	2.24	41.3	39.8	2.82	51.9	68.9	0.37	6.8	23.7	0	0	34.4	0	0	33	14.1	448	10.3	47.4	0.82
D-21	CCHF12	5.44	5.64	12.5	1.46	25.9	37.3	3.89	69	68.6	0.29	5.1	23	0	0	33.5	0	0	32.7	14	479	11.7	78.3	1.44
D-14	CCHF12	5.97	5.78	14.3	1.78	30.8	40.7	3.49	60.4	68.2	0.51	8.8	24	0	0	35.1	0	0	33.2	14.2	393	10.4	25.7	0.43
D-7	CCHF12	5.48	6.48	13.2	2.14	33	39	3.63	56	71.2	0.7	10.8	24.1	0.01	0.2	33.8	0	0	34.6	14.2	470	10.6	53.2	0.97
D0	CCHF12	5.75	5.77	14	1.88	32.6	40.2	3.11	53.9	69.9	0.78	13.5	24.3	0	0	34.8	0	0	33.1	13.9	386	10	45.4	0.79
D1	CCHF12	5.28	7.93	12.8	5.63	71	36.9	1.6	20.2	69.9	0.7	8.8	24.2	0	0	34.7	0	0	32.8	13.8	368	10	53.9	1.02
D3	CCHF12	5.44	4.19	13.3	1.4	33.5	37.8	2.59	61.8	69.5	0.19	4.5	24.4	0.01	0.2	35.2	0	0	32.3	13.5	375	10.1	23.9	0.44
D5	CCHF12	5.45	3.41	13.1	0.95	27.9	37.6	1.92	56.3	69	0.54	15.8	24	0	0	34.8	0	0	31.4	13.3	391	9.8	20.7	0.38
D7	CCHF12	4.72	3.03	11.4	0.94	31	32.6	1.85	61.1	69.1	0.24	7.9	24.2	0	0	35	0	0	31.2	13.3	348	9.3	36.3	0.77
D-70	CCHF13	6.55	9.19	14.9	6.02	65.5	46.1	2.37	25.8	70.4	0.8	8.7	22.7	0	0	32.3	0	0	35.2	15.1	379	10.4	27.5	0.42
D-63	CCHF13	6.07	8.45	13.9	6.08	72	43.4	1.92	22.7	71.5	0.45	5.3	22.9	0	0	32	0	0	35.8	14.9	412	10.7	111.7	1.84
D-56	CCHF13	6.27	6.09	14.3	3.06	50.3	45	2.7	44.3	71.8	0.33	5.4	22.8	0	0	31.8	0	0	37.3	15.7	440	10.8	81.5	1.3
D-49	CCHF13	6.12	6.17	14.1	3.14	50.9	44.2	2.6	42.1	72.2	0.43	7	23	0	0	31.9	0	0	38.3	16.2	472	10.9	68.5	1.12
D-42	CCHF13	6.38	5.86	14.6	2.51	42.7	45.5	2.81	48	71.3	0.52	8.9	22.9	0.01	0.2	32.1	0.01	0.2	37.5	16.2	405	10.7	51.7	0.81
D-35	CCHF13	6.24	6.68	14.3	2.42	36.2	45	3.6	53.9	72.1	0.66	9.9	22.9	0	0	31.8	0	0	38	16.1	399	10.3	66.8	1.07
D-28	CCHF13	6.36	7.11	14.6	3.58	50.4	45.4	2.92	41.1	71.4	0.6	8.4	23	0.01	0.1	32.2	0	0	37.5	15.8	455	10.8	57.2	0.9
D-21	CCHF13	6.05	6.02	14	2.26	37.6	43.7	3.39	56.3	72.2	0.37	6.1	23.1	0	0	32	0	0	36.7	14.9	449	10.7	98.6	1.63
D-14	CCHF13	6.35	12.07	14.9	8.33	69	45.4	2.86	23.7	71.5	0.86	7.1	23.5	0	0	32.8	0.02	0.2	36.8	15.5	435	10.8	72.4	1.14
D-7	CCHF13	6.11	7.44	14.3	4.44	59.6	44.7	2.37	31.9	73.2	0.63	8.5	23.4	0	0	32	0	0	37.5	14.9	420	10.5	113	1.85
D0	CCHF13	6.09	5.41	14.4	2.44	45.1	44.1	2.57	47.5	72.4	0.4	7.4	23.6	0	0	32.7	0	0	36.5	14.8	384	10.1	75.5	1.24
D1	CCHF13	5.78	12.68	13.7	9.98	78.7	42.4	2.07	16.3	73.4	0.63	5	23.7	0	0	32.3	0	0	36.8	14.7	372	9.8	98.8	1.71
D3	CCHF13	5.47	4.71	13	1.68	35.7	39.7	2.52	53.5	72.6	0.5	10.6	23.8	0.01	0.2	32.7	0	0	36.9	14.8	317	10.1	38.8	0.71
D5	CCHF13	5.47	2.74	13	0.97	35.4	39.8	1.42	51.8	72.8	0.35	12.8	23.8	0	0	32.7	0	0	35.8	14.4	268	10.4	12	0.22
D7	CCHF13	5.32	4.87	12.4	0.75	15.4	38.1	2.94	60.4	71.6	1.18	24.2	23.3	0	0	32.5	0	0	35.4	14.2	233	10.3	38.8	0.73
D-70	CCHF14	6	23.2	14.4	20.35	87.7	42.8	1.62	7	71.3	1.22	5.3	24	0.01	0	33.6	0	0	35.4	14.6	395	10.2	71.4	1.19
D-63	CCHF14	5.76	13.52	13.6	11.22	83	41.5	1.52	11.2	72	0.77	5.7	23.6	0.01	0.1	32.8	0	0	34.9	14.3	402	11.5	98.5	1.71
D-56	CCHF14	6.03	6.48	14.3	4.24	65.4	43.4	1.74	26.9	72	0.46	7.1	23.7	0.04	0.6	32.9	0	0	35.7	14.9	353	10.7	81.4	1.35
D-49	CCHF14	5.8	14.15	14	11.48	81.1	42.6	1.67	11.8	73.4	0.96	6.8	24.1	0.04	0.3	32.9	0	0	35.6	14.3	481	10.4	75.4	1.3
D-42	CCHF14	5.89	7.54	14.1	5.33	70.7	42.5	1.7	22.5	72.2	0.48	6.4	23.9	0.03	0.4	33.2	0	0	35.3	14.7	406	10.2	99.5	1.69
D-35	CCHF14	6.02	7.76	14.7	5.08	65.5	43.8	2.08	26.8	72.8	0.52	6.7	24.4	0.08	1	33.6	0	0	35.7	14.6	431	9.8	74	1.23
D-28	CCHF14	6.22	6.58	14.9	4.24	64.4	44.4	1.65	25.1	71.4	0.63	9.6	24	0.06	0.9	33.6	0	0	34.7	14.7	413	10	84	1.35
D-21	CCHF14	5.89	7.75	14.1	4.95	63.8	43	2.06	26.6	73	0.65	8.4	23.9	0.09	1.2	32.8	0	0	35.2	14.2	472	10.5	118.4	2.01
D-14	CCHF14	6.04	11.76	14.7	9.05	76.9	43.2	1.67	14.2	71.5	0.83	7.1	24.3	0.19	1.6	34	0.02	0.2	34.6	14.2	353	10.1	71.3	1.18
D-7	CCHF14	6.11	15.68	14.9	12.44	79.4	44.6	1.73	11	73	1.43	9.1	24.4	0.08	0.5	33.4	0	0	35.7	14.3	461	10	118.5	1.94
D0	CCHF14	6.32	13.05	15.3	10.94	83.8	45.2	1.24	9.5	71.5	0.85	6.5	24.2	0.02	0.2	33.8	0	0	34.6	14.1	404	9.9	77.7	1.23
D1	CCHF14	5.62	12.36	13.7	10.88	88.1	40.7	0.76	6.1	72.4	0.71	5.7	24.4	0.01	0.1	33.7	0	0	35.3	14.1	356	9.6	75.9	1.35
D3	CCHF14	5.58	5.61	13.6	3.36	59.9	40.4	1.68	29.9	72.4	0.48	8.6	24.4	0.09	1.6	33.7	0	0	35.3	14	268	10.2	45.2	0.81
D5	CCHF14	5.48	3.01	13.4	1.57	52.2	39.4	0.91	30.2	71.9	0.51	16.9	24.5	0.02	0.7	34	0	0	34	13.8	257	10	13.2	0.24
D7	CCHF14	5.2	5.53	12.4	2.3	41.6	37	2.17	39.2	71.2	1.05	19	23.8	0.01	0.2	33.5	0	0	33.6	13.7	250	10.7	26.5	0.51
D-70	CCHF15	6.08	10.23	13.7	7.16	69.9	41.4	2.42	23.7	68.1	0.65	6.4	22.5	0	0	33.1	0	0	35.4	15.4	393	9.6	65.7	1.08
D-63	CCHF15	6.03	7.68	13.5	4.99	65	42	2.24	29.2	69.7	0.43	5.6	22.4	0.01	0.1	32.1	0.01	0.1	36.7	15.8	396	10.5	123	2.04
D-56	CCHF15	5.97	6.13	13.5	2.93	47.8	41.2	2.76	45	69	0.44	7.2	22.6	0	0	32.8	0	0	35.7	15.4	342	10.3	85.4	1.43
D-49	CCHF15	6.02	10.36	13.7	6.59	63.6	42.3	3.15	30.4	70.3	0.62	6	22.8	0	0	32.4	0	0	36.5	15.6	430	10	90.9	1.51
D-42	CCHF15	6.19	5.73	14	2.42	42.2	42.5	2.95	51.5	68.7	0.36	6.3	22.6	0	0	32.9	0	0	35.4	15.6	399	9.9	90.4	1.46
D-35	CCHF15	5.95	6.3	13.5	2.33	37	41.8	3.56	56.5	70.3	0.41	6.5	22.7	0	0	32.3	0	0	36.6	15.4	414	9.4	95.2	1.6
D-28	CCHF15	6.25	5.21	14.5	2.75	52.8	43	2.08	39.9	68.8	0.34	6.5	23.2	0	0	33.7	0.04	0.8	35.6	15.5	503	10.6	100	1.6
D-21	CCHF15	6.05	6.95	13.6	3.15	45.4	42.5	3.47	49.9	70.2	0.33	4.7	22.5	0	0	32	0	0	36.6	15.3	434	10.3	108.3	1.79
D-14	CCHF15	6.18	9.7	14.4	5.6	57.7	42.5	3.49	36	68.8	0.57	5.9	23.3	0.03	0.3	33.9	0.01	0.1	35	14.9	400	9.8	72.9	1.18
D-7	CCHF15	6.16	6.93	14.1	3.62	52.3	43.4	2.9	41.8	70.5	0.4	5.8	22.9	0.01	0.1	32.5	0	0	36.6	15.4	474	9.8	111.5	1.81
D0	CCHF15	6.15	5.31	14.2	2.87	54	42.8	2.08	39.2	69.6	0.36	6.8	23.1	0	0	33.2	0	0	35.2	14.9	381	9.1	93.5	1.52
D1	CCHF15	5.94	5.68	13.7	3.96	69.7	41.7	1.49	26.2	70.2	0.22	3.9	23.1	0.01	0.2	32.9	0	0	35.6	14.9	346	9.3	70.1	1.18
D3	CCHF15	6.03	3.2	13.9	1.42	44.3	41.9	1.49	46.6	69.5	0.28	8.8	23.1	0.01	0.3	33.2	0	0	35.2	14.9	263	9.7	74.8	1.24
D5	CCHF15	5.77	5.18	13.3	1.11	21.5	40.2	2.96	57.1	69.7	1.11	21.4	23.1	0	0	33.1	0	0	35	14.7	281	9.1	79.6	1.38
D7	CCHF15	5.4	15.44	12.3	8.85	57.4	37.6	5.04	32.6	69.6	1.52	9.8	22.8	0.02	0.1	32.7	0.01	0.1	35	14.9	322	9.1	162	3
D-70	CCHF16	5.5	8.05	12.7	3.48	43.3	39.2	4.18	51.9	71.3	0.33	4.1	23.1	0.05	0.6	32.4	0.01	0.1	35.6	14.4	359	10.7	27	0.49
D-63	CCHF16	5.41	8.37	12.2	3.48	41.5	39	4.29	51.3	72.1	0.5	6	22.6	0.1	1.2	31.3	0	0	35.5	14.3	606	11.4	61.7	1.14
D-56	CCHF16	5.58	7.05	12.8	3.55	50.3	40.7	3.05	43.3	72.9	0.43	6.1	22.9	0.02	0.3	31.4	0	0	36.4	14.7	468	11	48.5	0.87
D-49	CCHF16	5.53	8.56	12.6	4.24	49.5	40.9	3.73	43.6	74	0.5	5.8	22.8	0.09	1.1	30.8	0	0	37	14.6	373	12	53.1	0.96
D-42	CCHF16	5.67	6.1	13	2.47	40.5	41.5	3.35	54.9	73.2	0.25	4.1	22.9	0.03	0.5	31.3	0	0	35.9	14.4	415	10.9	45.9	0.81
D-35	CCHF16	5.16	7.6	11.9	3.7	48.7	38.5	3.62	47.6	74.6	0.22	2.9	23.1	0.06	0.8	30.9	0	0	37.9	14.8	455	10.1	51.6	1
D-28	CCHF16	5.65	5.37	13.1	2.51	46.7	41.6	2.61	48.6	73.6	0.24	4.5	23.2	0.01	0.2	31.5	0	0	37	14.6	414	11.2	45.2	0.8
D-21	CCHF16	5.55	7.1	12.5	3.66	51.6	40.8	3.08	43.4	73.5	0.35	4.9	22.5	0.01	0.1	30.6	0	0	36.5	14.6	302	12.5	89.9	1.62
D-14	CCHF16	5.74	10.9	13.4	7.79	71.5	41.8	2.53	23.2	72.8	0.57	5.2	23.3	0	0	32.1	0.01	0.1	36.1	14.5	522	10.1	35.6	0.62
D-7	CCHF16	5.38	8.27	12.5	3.14	38	40.5	4.56	55.1	75.3	0.53	6.4	23.2	0.04	0.5	30.9	0	0	37.9	14.6	413	10.8	61.3	1.14
D0	CCHF16	5.6	8.1	13.2	5.04	62.3	41.2	2.82	34.8	73.6	0.23	2.8	23.6	0.01	0.1	32	0	0	36.8	14.7	253	10.8	45.9	0.82
D1	CCHF16	5.09	7.86	12.2	5.44	69.2	38.1	1.91	24.3	74.9	0.5	6.4	24	0.01	0.1	32	0	0	38.2	14.8	327	9.7	32.1	0.63
D3	CCHF16	5.21	4.24	12.5	1.23	28.9	38.7	2.33	55	74.3	0.63	14.9	24	0.05	1.2	32.3	0	0	37.1	14.5	333	10	40.1	0.77
D5	CCHF16	5.29	4.39	12.5	0.81	18.4	39.3	3.29	74.9	74.3	0.27	6.2	23.6	0.02	0.5	31.8	0	0	36.9	14.4	357	10	63	1.19
D7	CCHF16	4.93	5.77	11.6	2.37	41	36.3	2.59	44.9	73.6	0.8	13.9	23.5	0.01	0.2	32	0	0	36.1	14.5	435	9.2	45.8	0.93
D-70	CCHF17	5.74	5.82	12.8	2.39	41.1	39.8	3.02	51.9	69.3	0.41	7	22.3	0	0	32.2	0	0	38.1	16.2	350	9.5	31.6	0.55
D-63	CCHF17	5.55	6.81	12.5	3.16	46.4	39.2	3.05	44.8	70.6	0.6	8.8	22.5	0	0	31.9	0	0	39.2	16.2	434	10	72.7	1.31
D-56	CCHF17	5.8	6.8	13.1	3.17	46.6	40.7	3.15	46.3	70.2	0.48	7.1	22.6	0	0	32.2	0	0	39.2	16.4	392	10.3	65.5	1.13
D-49	CCHF17	5.75	6.97	13	3.35	48	41.2	2.89	41.5	71.7	0.73	10.5	22.6	0	0	31.6	0	0	40.4	16.7	424	10	43.1	0.75
D-42	CCHF17	6.01	6.44	13.7	2.85	44.2	42.7	3	46.6	71	0.59	9.2	22.8	0	0	32.1	0	0	39.8	16.8	395	10.4	69.1	1.15
D-35	CCHF17	5.61	6.51	12.7	2.7	41.5	40.3	3.29	50.5	71.8	0.52	8	22.6	0	0	31.5	0	0	40.9	16.9	361	10.4	60.6	1.08
D-28	CCHF17	6	5.83	13.7	2.2	37.8	42.7	3.01	51.6	71.2	0.62	10.6	22.8	0	0	32.1	0	0	39.4	16.5	420	10	43.8	0.73
D-21	CCHF17	5.95	6.23	13.4	2.46	39.5	42.7	3.07	49.3	71.8	0.7	11.2	22.5	0	0	31.4	0	0	38.4	15.4	280	11.2	54.7	0.92
D-14	CCHF17	5.9	6.49	14	3	46.3	42.1	2.91	44.8	71.4	0.58	8.9	23.7	0	0	33.3	0	0	38.2	15.7	422	9.6	43.1	0.73
D-7	CCHF17	5.61	6.25	13.3	2.92	46.7	41.1	2.68	42.9	73.3	0.65	10.4	23.7	0	0	32.4	0	0	38.3	15.2	451	10	94.2	1.68
D0	CCHF17	5.73	6.91	13.6	4.01	58.1	41.3	2.42	35	72.1	0.48	6.9	23.7	0	0	32.9	0	0	37.6	15.1	352	9.4	56.2	0.98
D1	CCHF17	5.43	6.59	13	4.16	63.1	39.8	1.89	28.7	73.3	0.54	8.2	23.9	0	0	32.7	0	0	37.8	15	382	9.4	51.6	0.95
D3	CCHF17	5.15	3.28	12.1	1.93	58.9	37.4	1.12	34.1	72.6	0.23	7	23.5	0	0	32.4	0	0	38.4	15.3	186	9.6	16.5	0.32
D3 (rerun)	CCHF17	5.12	3.21	12.2	1.89	58.8	37.2	1.1	34.3	72.7	0.22	6.9	23.8	0	0	32.8	0	0	38.1	15.3	196	10.1	17.9	0.35
D5	CCHF17	5.36	3.73	12.5	0.88	23.6	38.5	2.43	65.1	71.8	0.42	11.3	23.3	0	0	32.5	0	0	37.7	15	202	10.2	14.5	0.27
D7	CCHF17	5.06	16.67	11.7	1.07	6.4	36.3	11.91	71.4	71.7	3.68	22.1	23.1	0	0	32.2	0.01	0.1	37.9	15.2	268	9.9	6.1	0.12
D-70	CCHF18	6.72	15.15	14.5	5.61	37	43.7	8.63	57	65	0.89	5.9	21.6	0	0	33.2	0.02	0.1	34.8	17.4	380	11.1	26.9	0.4
D-63	CCHF18	6.5	13.21	14.1	4.52	34.2	42.8	7.93	60	65.8	0.76	5.8	21.7	0	0	32.9	0	0	33.6	16	386	12.4	39	0.6
D-56	CCHF18	6.43	12.78	14	4.97	38.9	42.9	6.83	53.4	66.7	0.98	7.7	21.8	0	0	32.6	0	0	34.1	16	348	11.5	39.9	0.62
D-49	CCHF18	6.28	16.4	13.7	8.05	49	42.6	7.39	45.1	67.8	0.95	5.8	21.8	0	0	32.2	0.01	0.1	34.7	15.7	421	11.8	48.4	0.77
D-42	CCHF18	6.35	11.44	13.9	3.22	28.1	42.5	7.25	63.4	66.9	0.97	8.5	21.9	0	0	32.7	0	0	34	16	360	11.8	59.1	0.93
D-35	CCHF18	6.02	11.44	13.2	3.42	29.9	41.6	7.18	62.8	69.1	0.84	7.3	21.9	0	0	31.7	0	0	35.2	15.4	396	10.6	67.4	1.12
D-28	CCHF18	6.34	14.51	13.9	8.41	57.9	42.9	5.29	36.5	67.7	0.81	5.6	21.9	0	0	32.4	0	0	34.6	15.1	384	11.9	40.6	0.64
D-21	CCHF18	5.93	10.92	12.8	3.52	32.2	40.1	6.79	62.2	67.6	0.61	5.6	21.6	0	0	31.9	0	0	33.5	14.9	298	13.3	59.3	1
D-14	CCHF18	6.55	10.55	14.5	4.66	44.1	43.2	5.26	49.9	66	0.62	5.9	22.1	0	0	33.6	0.01	0.1	33.2	15.7	414	11.2	34.1	0.52
D-7	CCHF18	5.98	13.92	13.3	5.72	41.1	41.3	6.97	50.1	69.1	1.23	8.8	22.2	0	0	32.2	0	0	34.4	14.8	404	11.2	59.8	1
D0	CCHF18	6.57	14.05	14.6	7.64	54.4	44.4	4.93	35.1	67.6	1.48	10.5	22.2	0	0	32.9	0	0	34	14.9	289	10.9	54.5	0.83
D1	CCHF18	5.92	8.39	13.2	2.69	32	40.6	4.52	53.9	68.6	1.18	14.1	22.3	0	0	32.5	0	0	34.3	14.7	276	11.3	51.5	0.87
D3	CCHF18	5.85	11.89	13.2	5.31	44.7	39.8	5.14	43.2	68	1.44	12.1	22.6	0	0	33.2	0	0	33.8	14.6	351	10.6	46.8	0.8
D5	CCHF18	5.68	8.3	12.8	1.93	23.2	38.7	5.17	62.3	68.1	1.2	14.5	22.5	0	0	33.1	0	0	33.9	14.6	242	10.8	58.5	1.03
D7	CCHF18	5.63	17.31	12.4	9.49	54.8	38	6.45	37.3	67.5	1.37	7.9	22	0	0	32.6	0	0	33.7	14.6	385	10	77.1	1.37
D-70	CCHF19	5.57	10.45	12.5	8.26	79.1	37.1	1.57	15	66.6	0.62	5.9	22.4	0	0	33.7	0	0	36.1	15.7	307	10.6	39	0.7
D-63	CCHF19	5.22	11.46	11.5	9.56	83.4	35.1	1.51	13.2	67.2	0.39	3.4	22	0	0	32.8	0	0	36.2	15.5	356	11.4	53.2	1.02
D-56	CCHF19	5.46	8.36	12.3	6.21	74.3	36.8	1.65	19.7	67.4	0.5	6	22.5	0	0	33.4	0	0	36.3	15.8	316	11.3	59	1.08
D-49	CCHF19	5.3	7.52	11.9	5.2	69.1	36.2	1.54	20.5	68.3	0.66	8.8	22.5	0.12	1.6	32.9	0	0	36.7	15.8	353	10.9	31.8	0.6
D-42	CCHF19	5.58	5.18	12.6	3.31	63.9	37.6	1.55	29.9	67.4	0.31	6	22.6	0.01	0.2	33.5	0	0	36.5	15.7	319	10.8	44.1	0.79
D-35	CCHF19	5.37	6.61	12	4.3	65	36.7	1.71	25.9	68.3	0.6	9.1	22.3	0	0	32.7	0	0	36.6	15.6	323	10.5	37.1	0.69
D-28	CCHF19	5.62	5.29	12.7	3.26	61.6	37.7	1.46	27.6	67.1	0.46	8.7	22.6	0.11	2.1	33.7	0	0	36.1	15.7	313	11.2	50.6	0.9
D-21	CCHF19	5.16	4.19	11.6	2.07	49.3	34.8	1.72	41.1	67.4	0.36	8.6	22.5	0.04	1	33.3	0	0	36	15.5	376	10.7	37.7	0.73
D-14	CCHF19	5.63	6.93	12.9	4.84	69.9	37.9	1.53	22.1	67.3	0.55	7.9	22.9	0.01	0.1	34	0	0	35.9	15.5	300	10.5	34.9	0.62
D-7	CCHF19	5.54	8.16	12.6	6.08	74.5	38.1	1.42	17.4	68.8	0.65	8	22.7	0.01	0.1	33.1	0	0	36.6	15.5	384	11.2	60.4	1.09
D0	CCHF19	5.58	10.71	12.8	8.54	79.7	37.7	1.36	12.7	67.6	0.76	7.1	22.9	0.05	0.5	34	0	0	36	15.4	314	9.9	49.7	0.89

D1	CCHF19	5.11	12.13	11.6	10.54	86.9	34.7	0.97	8	67.9	0.6	4.9	22.7	0.02	0.2	33.4	0	0	35.9	15.5	305	9.9	44.5	0.87
D3	CCHF19	4.97	3.49	11.4	1.95	55.9	33.2	1.32	37.8	66.8	0.18	5.2	22.9	0.04	1.1	34.3	0	0	35.5	15.3	255	10.2	30.3	0.61
D5	CCHF19	4.87	5.2	11.1	1.44	27.7	32.4	2.58	49.6	66.5	1.14	21.9	22.8	0.03	0.6	34.3	0.01	0.2	34.6	15	242	10.2	14.6	0.3
D7	CCHF19	4.86	12.52	11	3.55	28.4	32.4	7.98	63.7	66.7	0.97	7.7	22.6	0.01	0.1	34	0.01	0.1	35.3	15.3	263	10.5	53	1.09
D-70	CCHF20	5.74	6.77	12.8	4.74	70	38.8	1.78	26.3	67.6	0.25	3.7	22.3	0	0	33	0	0	34	14.8	291	12.1	48.8	0.85
D-63	CCHF20	5.41	7.34	11.9	4.77	65	37.4	2.14	29.2	69.1	0.39	5.3	22	0.04	0.5	31.8	0	0	34.5	14.5	298	12.8	54.1	1
D-56	CCHF20	5.5	7.65	12.3	5.21	68.1	37.6	2.03	26.5	68.4	0.38	5	22.4	0.03	0.4	32.7	0	0	33.9	14.5	263	12.2	55.6	1.01
D-49	CCHF20	5.42	7.14	12.1	5.01	70.2	37.7	1.75	24.5	69.6	0.35	4.9	22.3	0.02	0.3	32.1	0.01	0.1	35.2	14.9	288	12.1	75.3	1.39
D-42	CCHF20	5.62	6.38	12.6	4.35	68.2	39	1.61	25.2	69.4	0.4	6.3	22.4	0.02	0.3	32.3	0	0	34.7	14.7	295	12.3	85.4	1.52
D-35	CCHF20	5.49	3.94	12.2	2.03	51.5	38.9	1.56	39.6	70.9	0.35	8.9	22.2	0	0	31.4	0	0	36.1	14.7	306	11.6	59.8	1.09
D-28	CCHF20	5.66	6.23	12.8	4.6	73.8	39.6	1.35	21.7	70	0.27	4.3	22.6	0.01	0.2	32.3	0	0	34.7	14.7	287	12.4	53.2	0.94
D-21	CCHF20	5.48	5.12	12.3	2.82	55.1	38.8	1.9	37.1	70.8	0.37	7.2	22.4	0.03	0.6	31.7	0	0	35.3	14.4	163	14.9	72.9	1.33
D-21 (rerun)	CCHF20	5.5	5.22	12.3	2.9	55.6	39	1.85	35.4	70.9	0.46	8.8	22.4	0.01	0.2	31.5	0	0	35.1	14.4	188	14.8	74.3	1.35
D-14	CCHF20	5.7	7.4	13.1	5.46	73.8	39.6	1.48	20	69.5	0.36	4.9	23	0.09	1.2	33.1	0.01	0.1	34	14.3	196	13	62.7	1.1
D-14 (rerun)	CCHF20	5.7	6.83	13.1	5.07	74.2	39.8	1.35	19.8	69.8	0.32	4.7	23	0.08	1.2	32.9	0.01	0.1	34.2	14.3	174	12.2	63.3	1.11
D-7	CCHF20	5.43	6.02	12.6	4.28	71.1	38.9	1.28	21.3	71.6	0.44	7.3	23.2	0.02	0.3	32.4	0	0	35.5	14.3	302	12.4	76.6	1.41
D0	CCHF20	5.64	5.64	12.9	4.12	73	40.3	1.21	21.5	71.5	0.29	5.1	22.9	0.02	0.4	32	0	0	34.4	14	248	11.8	----	0.21
D0 (rerun)	CCHF20	5.74	5.85	13.3	4.3	73.5	40	1.15	19.7	69.7	0.38	6.5	23.2	0.02	0.3	33.3	0	0	33.5	14.2	253	11.9	26.4	0.46
D1	CCHF20	5.01	8.6	11.7	7.15	83.2	35.8	1	11.6	71.5	0.45	5.2	23.4	0	0	32.7	0	0	34.3	14	251	11.1	47.6	0.95
D3	CCHF20	5.67	4.53	13.1	2.81	62	40.2	1.56	34.4	70.9	0.13	2.9	23.1	0.03	0.7	32.6	0	0	34.5	14.1	245	11.7	56.7	1
D5	CCHF20	5.15	1.34	12.1	0.45	33.6	36.1	0.65	48.5	70.1	0.23	17.2	23.5	0.01	0.7	33.5	0	0	33.3	14	169	11.2	37.1	0.72
D5 (rerun)	CCHF20	5.21	1.36	12	0.44	32.4	36.6	0.56	41.2	70.2	0.35	25.7	23	0.01	0.7	32.8	0	0	33.4	13.9	177	12.2	36.5	0.7
D7	CCHF20	5.37	2.91	12.3	0.79	27.1	36.9	1.81	62.2	68.7	0.31	10.7	22.9	0	0	33.3	0	0	32.5	13.7	78	12.6	6.4	0.12
D7 (rerun)	CCHF20	5.46	3.05	12.6	0.81	26.6	37.1	1.93	63.3	67.9	0.3	9.8	23.1	0.01	0.3	34	0	0	32.1	13.8	73	16	5.5	0.1
D7 (rerun)	CCHF20	5.4	2.98	12.7	0.78	26.1	36.3	1.93	64.8	67.2	0.27	9.1	23.5	0	0	35	0	0	31.5	13.7	75	15.8	10.3	0.19

Supplemental Table 3: Complete Histological Findings for Study 2

	CCHF09	CCHF10	CCHF11	CCHF12	CCHF13	CCHF14	CCHF15	CCHF16	CCHF17	CCHF18	CCHF19	CCHF20
	Sham						repNP + repGPC					
**Skin: Inj site**												
lymphohistiocytic dermatitis	0	1	0	1	0	0	0	0	0	0	0	0
**ALN**												
	0	0	np	0	np	0	0	0	0	0	0	0
**ILN**												
	0	0	0	0	0	0	0	0	0	0	0	0
**Salivary gland**												
lymphoplasmacytic infiltrates	0	1	0	1	1	1	0	2	0	0	0	0
**RLL**												
lymphocytic perivascular cuffing	0	0	0	0	0	0	0	0	2	0	0	0
**Heart**												
lymphohistiocytic infiltrates	0	1	1	1	0	2	2	0	0	0	0	0
**Liver**												
Hepatocellular necrosis	1	1	2	1	1	1	0	0	0	0	0	0
Portal inflammation	0	1	1	1	1	1	1	1	1	0	0	0
Lymphohistiocytic nodules	1	0	3	2	3	3	1	0	1	0	1	1
Ito cell hyperplasia	2	0	2	0	2	2	2	0	0	0	3	3
microthrombi	0	0	0	0	0	0	0	0	1	0	0	0
**Spleen**												
Lymphoid depletion	0	0	0	0	0	0	0	0	0	0	0	0
Lymphoid hyperplasia	0	0	0	0	0	0	0	0	0	0	0	0
Parafollicular fibrin	0	0	0	0	0	0	0	0	0	0	0	0
**Kidney**												
Tubular epithelial degeneration/necrosis and sloughing	0	0	0	0	0	0	0	0	0	0	0	0
Neutrophilic interstitial nephritis	0	0	0	0	0	0	0	0	0	0	0	0
Lymphocytic interstitial nephritis	0	0	0	0	0	0	1	0	0	0	0	0
**Adrenal gland**												
	0	0	0	0	0	0	0	0	0	0	0	0
**Pancreas**												
	0	0	0	0	0	0	0	0	0	0	0	0
**Mes LN**												
granuloma	np	0	0	1	0	0	0	1	0	0	0	0
hemosiderin-laden macrophages	0	0	0	0	0	0	0	0	1	0	2	1
**Stomach**												
lymphoplamacytic infiltrate	3	2	3	0	3	3	0	3	0	1	0	0
**Duodenum**												
lymphoplamacytic infiltrate	2	2	0	0	2	2	3	2	2	2	0	1
**Jejunum**												
ascarid granuloma	0	0	0	1	0	0	0	0	0	0	0	0
**Ileum**												
lymphoplamacytic infiltrate	2	0	0	0	0	0	0	0	0	0	0	0
villi loss and atrophy	1	0	0	0	0	0	0	0	0	0	0	0
**Cecum**												
lymphoplamacytic infiltrate	0	1	0	0	0	0	0	1	1	1	1	1
**Colon**												
lymphoplamacytic infiltrate	0	0	0	0	0	0	0	0	1	0	0	0
**Urinary bladder**												
subepithelial lymphocytic infiltrate	0	0	0	0	0	0	0	0	2	0	0	0
Repro												
**ovary**												
	0	na	0	na	0	0	0	na	0	na	0	0
**Testicel**												
	na	0	na	0	na	na	na	0	na	0	np	np
**Bone marrow**												
brown granular pigment	0	np	0	0	0	np	2	np	0	np	0	0
**CLN**												
	0	0	0	0	0	0	0	0	np	0	0	0
**Misc**												
						*					*	

na = not applicable
np = not present

0 = No lesions
1 = Minimal (1-10%)
2 = Mild (11-25%)
3 = Moderate (26-50%)
4 = Marked (51-75%)
5 = Severe (76-100%)

0 = No lesions
1 = Minimal (1-10%)
2 = Mild (11-25%)
3 = Moderate (26-50%)
4 = Marked (51-75%)
5 = Severe (76-100%)

0 = No lesions
1 = Minimal (1-10%)
2 = Mild (11-25%)
3 = Moderate (26-50%)
4 = Marked (51-75%)
5 = Severe (76-100%)

Supplemental Table 4: Complete IHC Findings for Study 2

	CCHF09	CCHF10	CCHF11	CCHF12	CCHF13	CCHF14	CCHF15	CCHF16	CCHF17	CCHF18	CCHF19	CCHF20
	Sham						repNP + repGPC					
Heart												
	0	0	0	0	0	0	0	0	0	0	0	0
Liver												
kupffer cells, endothelial cells and hepatocytes	4	2	3	3	3	3	0	0	0	0	0	4
Spleen												
endothelial cells and macrophages	1	2	2	3	3	4	0	0	0	0	0	3
Adrenal gland												
endothelial cells and macrophages	1	1	1	1	2	2	0	0	0	0	0	2

IHC attachment
0=none
1=rare/few
2=scattered
3=moderate
4=numerous
5=diffuse
